# Predictors of residual antimalarial drugs in the blood in community surveys in Tanzania

**DOI:** 10.1371/journal.pone.0202745

**Published:** 2018-09-07

**Authors:** Joanna Gallay, Emilie Pothin, Dominic Mosha, Erick Lutahakana, Festo Mazuguni, Martin Zuakulu, Laurent Arthur Decosterd, Blaise Genton

**Affiliations:** 1 Department of Epidemiology and Public Health, Swiss Tropical and Public Health Institute, Basel, Switzerland; 2 University of Basel, Basel, Switzerland; 3 Service and Laboratory of Clinical Pharmacology, University Hospital, Lausanne, Switzerland; 4 Ifakara Health Institute, Dar es Salaam, Tanzania; 5 Division of Infectious Diseases and Department of Community Health, University Hospital, Lausanne, Switzerland; Tulane University School of Public Health and Tropical Medicine, UNITED STATES

## Abstract

**Background:**

Understanding pattern of antimalarials use at large scale helps ensuring appropriate use of treatments and preventing the spread of resistant parasites. We estimated the proportion of individuals in community surveys with residual antimalarials in their blood and identified the factors associated with the presence of the most commonly detected drugs, lumefantrine and/or desbutyl-lumefantrine (LF/DLF) or sulfadoxine-pyrimethamine (SP).

**Methods:**

A cross-sectional survey was conducted in 2015 in three regions of Tanzania with different levels of malaria endemicity. Interviews were conducted and blood samples collected through household surveys for further antimalarial measurements using liquid chromatography coupled to tandem mass spectrometry. In addition, diagnosis and treatment availability was investigated through outlet surveys. Multilevel mixed effects logistic regression models were used to estimate odds ratios for having LF/DLF or SP in the blood.

**Results:**

Amongst 6391 participants, 12.4% (792/6391) had LF/DLF and 8.0% (510/6391) SP in the blood. Factors associated with higher odds of detecting LF/DLF in the blood included fever in the previous two weeks (OR = 2.6, p<0.001), living in districts of higher malaria prevalence (OR = 1.5, p<0.001) and living in a ward in which all visited drug stores had artemisinin-based combination therapies in stocks (OR = 2.7, p = 0.020). Participants in older age groups were less likely to have LF/DLF in the blood (OR = 0.9, p<0.001). Factors associated with higher odds of having SP in the blood included being pregnant (OR = 4.6, p<0.001), living in Mwanza (OR = 3.9, p<0.001 compared to Mbeya), fever in the previous two weeks (OR = 1.7, p<0.001) and belonging to older age groups (OR = 1.2, p<0.001).

**Conclusion:**

The most significant predictors identified were expected. History of fever in the past two weeks and young age were significant predictors of LF/DLF in the blood, which is encouraging. Antimalarial drug pressure was high and hence the use of recommended first-line drugs in combination with malaria Rapid Diagnostics Tests should be promoted to ensure appropriate treatment.

## Introduction

Artemisinin-based combination therapies (ACTs) are the most potent weapon in treating falciparum malaria [1], representing the second highest impact intervention on malaria control between 2000 and 2015 [2]. In response to the development of parasite resistance to conventional antimalarials such as chloroquine, the World Health Organisation (WHO) recommended ACTs as first-line therapy for uncomplicated malaria in 2006 [3] when the Ministry of Health and Social Welfare of Tanzania had already recommended artemether-lumefantrine (Alu) as standard therapy in 2005 [4]. The use of antimalarials as combination therapy is yet clearly of benefit for the individuals [5], but artemisinin itself is rapidly eliminated while the “partner drugs” persist in the bloodstream as monotherapy for weeks due to their long half-lives [6]. Despite its poor efficacy and a high proportion of resistant parasites in Tanzania [7–9], sulfadoxine-pyrimethamine (SP) is still widely used as self-medication outside of intermittent preventive treatment during pregnancy (IPTp) [10,11]. The antimalarials currently used in combination with artemisinin derivates in Tanzania as well as sulfadoxine (SD) and pyrimethamine (PM) have long half-lives [lumefantrine (LF): 3 to 6 days, its active metabolite desbutyl-lumefantrine (DLF): 5 to 6 days, amodiaquine (AQ): 2.7–5.1 hours; its active metabolite N-desethyl-amodiaquine (DAQ): 7 to 10 days; quinine (Q): 7 to 21 hours; chloroquine (CQ): 10 to 30 days; mefloquine (MQ): 17 to 24 days; SD: 5 to 7 days; PM: 3 to 4 days)] [12–17]. The extensive and often inadequate use of these long-acting compounds constitutes a critical factor contributing to drug pressure [18], which in turn is a potent force selecting resistant parasites [19,20]. Thus, correct use of antimalarials is now one of the five goals and recommendations of WHO Global plan for artemisinin resistance containment [1]. Understanding the pattern of antimalarial drugs usage will help designing specific and targeted interventions aimed at reducing drug pressure and monitoring the implementation of diagnostic and treatment policies.

Previous studies have explored the effect of environmental and behavioral factors on population drug use. Most of them relied on questionnaires [10,21–23] although there was no indication that information collected through self-reporting was reliable. With such methodology, uptake of antimalarials was reported to be low [20,24]. On the other hand, studies conducted during the CQ or SP eras [19,20,25,26] using biochemical markers in addition to questionnaires showed high prevalence of individuals with detectable drug in urine or blood, although the tests used had a rather poor sensitivity. Two recent studies using liquid chromatography coupled to tandem mass spectrometry (LC-MS/MS), a sensitive analytical method, showed that 52% of the patients upon admission to a health facility (HF) in Cambodia [27] and 74% in Tanzania [28] had detectable antimalarials in their blood, although all of them stated that they had not taken any drug in the previous four weeks. These observations indicate frequent recourse to self-medication and poor reliability of self-reporting when assessing drug use. Our study first aimed at evaluating the actual state of population drug pressure by estimating the proportion of individuals in community surveys with residual antimalarials in their blood, and then at identifying the factors associated with the presence of LF or SP in the blood.

## Methods

A cross-sectional investigation including household-based and drug outlet-based surveys was conducted concurrently. Detailed study setting, population and sampling have been described elsewhere [29] and can be summarized as follows.

### Study setting and population

Surveys were conducted in three regions of Tanzania with different levels of malaria endemicity, i.e moderately high for Mtwara and Mwanza (17.4% and 16.1% respectively) and low for Mbeya (2.3%) [30]. Our study took place from May to August 2015 following the rainy season. In this country where the population is predominantly rural, over 95% of inhabitants of mainland Tanzania are at risk of malaria [31]. Alu is the first line recommended therapy in Tanzania and is free of charge in public health facilities (HFs) for under five children, pregnant women, elderly and those who cannot afford to pay [30]. Although no-longer indicated for malaria case management, SP is still widely available both in the private and public sector, according to recent outlet surveys [32].

### Study sampling and data collection

Tanzania has four different administrative levels: regions, divisions, districts and wards (which include five to seven villages). In each region, one urban and two rural districts were selected. Three wards per district, four streets or sub villages per ward, 20 household per sub village/street and up to six individuals per household were randomly selected. Wards were randomly selected proportionally to their population size amongst all the wards included in the selected districts, using an adapted version of sample command in R (version 3.4.0), using population as probability weights. The same methodology was applied for the villages and street selection. The questionnaire in Swahili was divided into 3 parts: i) household-related questions on indoor residual spraying (IRS), time to the closest HF and closest drug retailer; ii) demographic information, use of bed nets, pregnancy, history of fever in the previous two weeks and history of antimalarial use in the previous month; iii) information on health seeking behaviour. The drug outlet surveys included all HFs (small district hospitals, public and private health centres, dispensaries) and drug stores [pharmacies, registered accredited drug dispensing outlets (ADDOs) and non-registered drug retailers, general stores and kiosks] surrounding the selected villages. Diagnostic tools [malaria Rapid Diagnostics Tests (mRDTs) and microscope] and antimalarials in stocks at the time of the visit were recorded.

### Laboratory procedures

Capillary blood was collected from all participants of the household surveys for mRDTs analysis (ParaHIT-f test, Span diagnostic Ltd, Surat, India, detecting HRP-2 antigens) and applied on filter paper cards (FTA DMPK-B cards, Whatman, GE Healthcare). These were able to dry at room temperature for at least two hours before being placed in a specific bag with desiccant and stored in a -10°C freezer at the end of the day at study site. Samples were then transferred to a -80°C freezer at the Ifakara Health Institute within one month and finally at the University Hospital of Lausanne, Switzerland. The analysis of seven antimalarials and two active metabolites (LF, DLF, SD, PM, Q, MQ, CQ, AQ, DAQ) was further performed by LC-MS/MS in the dried blood spots (DBS) samples. The analytical method achieved good performances in terms of trueness (-12.1 to +11.1%) and precision (1.4 to 15.0%). All analytes were stable in DBS kept for 24h at room temperature and at 37°C [33,34]. They were also found to be stable when mimicking the study storage and transportation conditions (unpublished data).

### Data management and statistical analysis

The Open Data Kit collection tool (ODK) programmed on electronic tablets was used to collect the data and record Global Positioning System coordinates of each household and drug outlet visited. Data was stored on the ODK Aggregate data repository at the end of each survey day. R (version 3.4.0) was used for data cleaning and management and to produce logistic regressions as well as forest plots using the lme4 and sjPlot packages.

#### Variable definitions

Individuals were considered having an antimalarial in the blood if the concentrations measured in their corresponding DBS sample were equal or higher than the lower limit of quantification (LOQ) [34]. Age was categorized as presented in Tables 1 and 2, based on the age distribution of the sampled population. Mean malaria endemicity was classified according to the most recent epidemiological profile of mainland Tanzania from the last Malaria Indicator Survey [35]. The following variables were used as binary variables: history of fever (in the two weeks prior to the survey), mRDT result (performed on the day of the survey), bednet use (the previous night), IRS (the previous year), pregnancy (at the time of the survey) and living in an urban or rural district. Participants were asked to estimate the time to reach the closest HF or drug store (DS) using the mode of transport they would normally use. Outlets were considered to have mRDTs and antimalarials in stock if at least one non-expired test and respectively one complete non-expired treatment of any antimalarial for any age/weight group was observed.

**Table 1:** Sample characteristics of included individuals and strength of association between variables and presence of LF/DLF in the blood.

	Total participants	Participants with LF/DLF in the blood					
	N (%)	N (%)	95% CI	Bivariate analysis		Multivariate analysis	
				ORs	*p*-value	ORs	*p*-value
**Total**	**6391** (100.0)	**792** (12.4)	11.7–13.1	-	-	-	-
Sex							
Male	2802 (43.8)	350 (12.5)	11.5–13.5	0.97	0.673	-	-
Female	3573 (55.9)	441 (12.3)	11.4–13.2				
Missing	16 (0.3)	-	-				
**Age**							
0–4 years	1135 (17.8)	157 (13.8)	12.1–15.5	0.92	<0.001	0.92	<0.001
5–9 years	996(15.6)	170 (17.1)	15.1–19.0				
10–14 years	765 (12.0)	111 (14.5)	12.4–16.6				
15–24 years	973 (15.2)	83 (8.5)	7.1–10.0				
25–44 years	1422 (22.2)	129 (9.1)	7.8–10.3				
45–59 years	552 (8.6)	79 (14.3)	11.9–16.8				
60–100 years	445 (7.0)	49 (11.0)	8.6–13.5				
Missing	103 (1.6)	-	-				
**Pregnant**							
Yes	104 (1.6)	10 (9.6)	4.8–14.4	0.71	0.297	-	-
No	1569 (24.6)	135 (8.6)	7.4–9.8				
Not applicable	4718 (73.8)	-	-				
**Had a fever in the previous 2 weeks**							
Yes	1021 (16.0)	247 (24.2)	22.0–26.4	2.70	<0.001	2.62	<0.001
No	5364 (83.9)	544 (10.1)	9.5–10.8				
Missing	6 (0.1)	-	-				
**mRDT result**							
Positive	1117 (17.5)	222 (19.9)	17.9–21.8	1.25	0.023	1.14	0.183
Negative	5272 (82.5)	570 (10.8)	10.1–11.5				
Missing	2 (0.0)	-	-				
**Net use**							
Yes	4180 (65.4)	555 (13.3)	12.5–14.2	1.36	<0.001	1.30	0.004
No	2211 (34.6)	238 (10.8)	9.7–11.8				
Missing	0 (0.0)	-	-				
**IRS in the previous year**							
Yes	1073 (16.8)	147 (13.7)	12.0–15.4	0.99	0.802	-	-
No	5316 (83.2)	645 (12.1)	11.4–12.9				
Missing	2 (0.0)	-	-				
**Area**							
Urban	2185 (34.2)	172 (7.9)	6.9–8.8	1.59	0.359	-	-
Rural	4206 (65.8)	620 (14.7)	13.8–15.7				
Missing	0 (0.0)	-	-				
**Region**							
Mbeya (reference)	1970 (30.8)	151 (7.7)	6.7–8.6	-	-	-	-
Mwanza	2304 (36.1)	338 (14.7)	13.5–15.9	2.15	0.137	-	-
Mtwara	2117 (33.1)	303 (14.3)	13.1–15.6	2.49	0.075	-	-
Missing	0 (0.0)	-	-				
**PfPR_2-10_ of the ward**							
0 < 1%	1421 (22.2)	244 (17.2)	15.5–18.8	0.97	0.655	-	-
1 - < 5%	0 (0.0)	-	-				
5 - < 10%	1453 (22.7)	202 (13.9)	12.4–15.4				
10 - < 50%	2062 (32.3)	183 (8.9)	7.8–9.9				
> 50%	1455 (22.8)	163 (11.2)	9.8–12.6				
Missing	0 (0.0)	-	-				
**PfPR_2-10_ of the district**							
0 < 1%	1244 (19.5)	48 (3.9)	3.0–4.8	1.53	0.002	1.52	<0.001
1 - < 5%	0 (0.0)	-	-				
5 - < 10%	1470 (23.0)	138 (9.4)	8.1–10.6				
10 - < 50%	2259 (35.3)	343 (15.2)	13.9–16.4				
> 50%	1418 (22.2)	263 (18.6)	16.8–20.2				
Missing	0 (0.0)	-	-				
Time to the closest HF*							
<15 min	2134 (33.4)	276 (12.9)	11.7–14.1	0.85	0.011	0.84	0.007
15 min to 1h	3483 (54.5)	430 (12.3)	11.4–13.3				
1h to 2h	564 (8.8)	57 (10.1)	8.0–12.2				
> 2h	167 (2.6)	26 (15.6)	10.9–20.2				
Don’t know	43 (0.7)	-	-				
Time to the closest DS**							
<15 min	2911 (45.5)	331 (11.4)	10.4–12.3	1.05	0.385	-	-
15 min to 1h	2599 (40.7)	354 (13.6)	12.5–14.7				
1h to 2h	461 (7.2)	60 (13.0)	10.4–15.6				
> 2h	63 (1.0)	9 (14.3)	7.0–21.5				
Don’t know	357 (5.6)	-	-				
**ACTs in stocks in all the visited HF of the ward**							
Yes	6391 (100.0)	792 (12.4)	11.7–13.1	-	-	-	-
No	0 (0.0)	-	-				
Missing	0 (0.0)	-	-				
AM*** **in stocks in all the visited HF of the ward**							
Yes	6391 (100.0)	792 (12.4)	11.7–13.1	-	-	-	-
No	0 (0.0)	-	-				
Missing	0 (0.0)	-	-				
**mRDTs in stocks in all the visited HF of the ward**							
Yes	4986 (78.0)	534 (10.7)	10.0–11.4	0.68	0.139	-	-
No	1405 (22.0)	258 (18.4)	16.7–20.1				
Missing	0 (0.0)	-	-				
**ACTs in stocks in all the visited DS of the ward**							
Yes	5528 (86.5)	667 (12.1)	11.3–12.8	2.88	0.008	2.69	0.020
No	399 (6.2)	16 (4.0)	2.4–5.6				
Missing	464 (7.3)	-	-				
**AM in stocks in all the visited DS of the ward**							
Yes	5694 (89.1)	669 (11.7)	11.0–12.4	2.53	0.039	-	-
No	233 (3.6)	14 (6.0)	3.4–8.6				
Missing	464 (7.3)	-	-				
**mRDTs in stocks in all the visited DS of the ward**							
Yes	713 (11.2)	56 (7.9)	6.2–9.5	0.73	0.126	-	-
No	5217 (81.6)	627 (12.0)	11.3–12.8				
Missing	464 (7.3)	-	-				

*HF = Health Facility.

**DS = Drug Store.

***AM = antimalarial drug.

In the bivariate analysis, p-values are based on log-likelihood ratio tests. Variables with more than two categories were considered as ordinal, except for the regions. Net use was defined as use in the previous night and PfPR_2-10_ as malaria endemicity.

**Table 2:** Sample characteristics of included individuals and strength of association between variables and presence of SP in the blood.

	Total participants	Participants with SP in the blood					
	N (%)	N (%)	95% CI	Bivariate analysis		Multivariate analysis	
				ORs	*p*-value	ORs	*p*-value
**Total**	**6391** (100.0)	**510** (8.0)	7.4–8.5	-	-		
**Sex**							
Male	2802 (43.8)	200 (7.1)	6.3–7.9	0.97	0.036	-	-
Female	3573 (55.9)	310 (8.7)	7.9–9.4				
Missing	16 (0.3)	-	-				
**Age**							
0–4 years	1135 (17.8)	67 (5.9)	4.7–7.1	1.21	<0.001	1.18	<0.001
5–9 years	996(15.6)	43 (4.3)	3.3–5.4				
10–14 years	765 (12.0)	41 (5.4)	4.0–6.7				
15–24 years	973 (15.2)	83 (8.5)	7.1–10.0				
25–44 years	1422 (22.2)	168 (11.8)	10.4–13.2				
45–59 years	552 (8.6)	62 (11.2)	9.0–13.4				
60–100 years	445 (7.0)	39 (8.8)	6.6–11.0				
Missing	103 (1.6)	-	-				
**Pregnant**							
Yes	104 (1.6)	28 (26.9)	19.7–34.1	4.91	<0.001	4.60	<0.001
No	1569 (24.6)	166 (10.6)	9.3–19.9				
Not applicable	4718 (73.8)	-	-				
**Had a fever in the previous 2 weeks**							
Yes	1021 (16.0)	127 (12.4)	10.7–14.1	1.58	<0.001	1.71	<0.001
No	5364 (83.9)	383 (7.1)	6.6–7.7				
Missing	6 (0.1)	-	-				
**mRDT result**							
Positive	1117 (17.5)	26 (2.3)	1.6–3.1	0.26	<0.001	0.28	<0.001
Negative	5272 (82.5)	484 (9.2)	8.5–9.8				
Missing	2 (0.0)	-	-				
**Net use**							
Yes	4180 (65.4)	356 (8.5)	7.8–9.2	1.07	0.541	-	-
No	2211 (34.6)	154 (7.0)	6.1–7.9				
Missing	0 (0.0)	-	-				
**IRS in the previous year**							
Yes	1073 (16.8)	82 (7.6)	6.3–9.0	0.44	<0.001	0.46	<0.001
No	5316 (83.2)	428 (8.0)	7.4–8.7				
Missing	2 (0.0)	-	-				
**Area**							
Urban	2185 (34.2)	224 (10.2)	9.2–11.3	0.57	0.145	-	-
Rural	4206 (65.8)	286 (6.8)	6.2–7.4				
Missing	0 (0.0)	-	-				
**Region**							
Mbeya (reference)	1970 (30.8)	134 (6.8)	5.9–7.7				
Mwanza	2304 (36.1)	281 (12.2)	11.1–13.3	1.59	0.241	3.92	<0.001
Mtwara	2117 (33.1)	95 (4.5)	3.7–5.2	0.56	0.156	1.02	0.942
Missing	0 (0.0)	-	-				
**PfPR_2-10_ of the ward**							
0 < 1%	1421 (22.2)	131 (9.2)	8.0–10.5	0.89	0.102	-	-
1 - < 5%	0 (0.0)	-	-				
5 - < 10%	1453 (22.7)	107 (7.4)	6.2–8.5				
10 - < 50%	2062 (32.3)	169 (8.2)	7.2–9.2				
> 50%	1455 (22.8)	103 (7.1)	6.0–8.2				
Missing	0 (0.0)	-	-				
**PfPR_2-10_ of the district**							
0 < 1%	1244 (19.5)	96 (7.7)	6.5–9.0	0.73	0.044	0.77	0.010
1 - < 5%	0 (0.0)	-	-				
5 - < 10%	1470 (23.0)	175 (11.9)	10.5–13.3				
10 - < 50%	2259 (35.3)	191 (8.5)	7.5–9.4				
> 50%	1418 (22.2)	48 (3.4)	2.6–4.2				
Missing	0 (0.0)	-	-		-		
Time to the closest HF*							
<15 min	2134 (33.4)	194 (9.1)	8.1–10.1	0.83	0.003	0.85	0.031
15 min to 1h	3483 (54.5)	279 (8.0)	7.2–8.8				
1h to 2h	564 (8.8)	27 (4.8)	3.3–6.3				
> 2h	167 (2.6)	8 (4.8)	2.1–7.5				
Don’t know	43 (0.7)	-	-				
Time to the closest DS**							
<15 min	2911 (45.5)	264 (9.1)	8.2–10.0	0.89	0.105	-	-
15 min to 1h	2599 (40.7)	200 (7.7)	6.8–8.6				
1h to 2h	461 (7.2)	36 (7.8)	5.7–9.9				
> 2h	63 (1.0)	3 (4.8)	0.3–9.2				
Don’t know	357 (5.6)	-	-				
ACTs in stocks in all the visited HF* of the ward							
Yes	6391 (100.0)	510 (8.0)	7.4–8.5	-	-	-	-
No	0 (0.0)	-	-				
Missing	0 (0.0)	-	-				
AM*** **in stocks in all the visited HF of the ward**							
Yes	6391 (100.0)	510 (8.0)	7.4–8.5	-	-	-	-
No	0 (0.0)	-	-				
Missing	0 (0.0)	-	-				
mRDTs in stocks in all the visited HF* **of the ward**							
Yes	4986 (78.0)	454 (9.1)	8.4–9.8	1.48	0.238	-	-
No	1405 (22.0)	56 (4.0)	3.1–4.8				
Missing	0 (0.0)	-	-				
**ACTs in stocks in all the visited DS of the ward**							
Yes	5528 (86.5)	467 (8.4)	7.8–9.1	1.27	0.839	-	-
No	399 (6.2)	17 (4.3)	2.6–5.9				
Missing	464 (7.3)	-	-				
AM in stocks in all the visited DS** **of the ward**							
Yes	5694 (89.1)	479 (8.4)	7.8–9.0	1.39	0.875	-	-
No	233 (3.6)	5 (2.1)	0.6–3.7				
Missing	464 (7.3)	-	-				
mRDTs in stocks in all the visited DS** **of the ward**							
Yes	713 (11.2)	96 (13.5)	11.4–15.6	1.73	0.223	-	-
No	5217 (81.6)	388 (7.4)	6.8–8.0				
Missing	464 (7.3)	-	-				

*HF = Health Facility.

**DS = Drug Store.

***AM = antimalarial drug.

#### Multilevel mixed effects logistic regression analysis

The presence of lumefantrine and/or desbutyl-lumefantrine (LF/DLF) and the presence of sulfadoxine and/or pyrimethamine (SP) were selected as outcome variables, as they were the antimalarials most frequently detected in the sampled population. LF and DLF were grouped as a unique outcome because the presence of either the parent compound (LF), the metabolite (DLF) or both of them concurrently in the blood indicates a recent intake of LF. A bivariate analysis was first performed to select variables based on the p-value of the log-likelihood ratio tests, with a cut-off of 0.2 [36]. This approach helps to first identify the most relevant variables to be included in a more complex model by minimizing the risk of selecting confounding factors. Multilevel mixed effects logistic regression models were then performed by a backward step-by-step procedure. Independent variables and their interactions were retained when significance level was less or equal to 0.05 and if they showed a model fit improvement [i.e. reduction in the Akaike Information Criteria (AIC) value of the mode]. According to sampling design, regions, district and wards were included as nested random effects.

### Ethical approval

This epidemiological study received approval from the Swiss Ethics Committees on research involving humans and from two responsible local authorities (Institutional review board of the Ifakara Research Institute and the National Institute for Medical Research in Tanzania). Interviews were conducted and capillary blood samples were obtained after receiving written informed consent in Kiswahili by the participants or their responsible caretaker.

## Results

In total, 6485 participants were included in the household surveys but 94 were excluded of the present analysis because their DBS sample was not found or mislabelled. On average, 237 individuals were interviewed in each ward, ranging from 166 to 380. The outlet surveys included 2 hospitals, 19 health centres, 39 dispensaries, 78 ADDOs, 57 non registered drug stores, 9 pharmacies and 4 general stores or kiosks.

### Site and study participants description

Females were 55.9% (3573/6391) while males were 43.8% (2802/6391). The median age was 17 years and ranged from three months, one of the inclusion criteria, to 100 years. Overall, 4421 participants were sampled in regions of high endemicity (2304 in Mwanza and 2117 in Mtwara) and 1970 in Mbeya, the region of low endemicity. Majority of the participants lived in rural districts [65.8% (4206/6391)]. Summary statistics for all assessed variables are shown in Tables 1 and 2.

### Prevalence of antimalarials in the blood of the surveyed population

**Fig 1:**
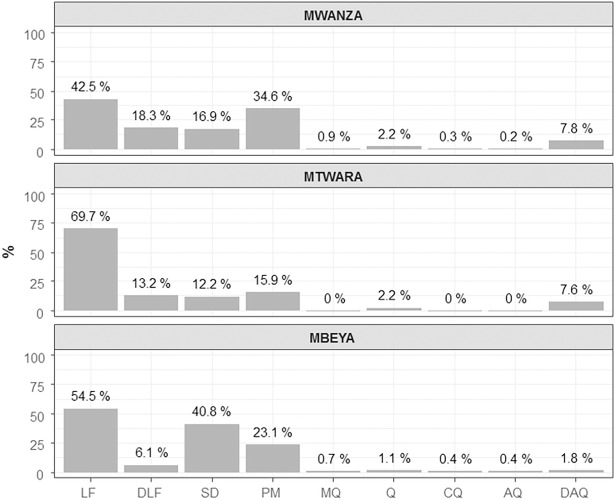
Percentage of participants with residual antimalarial concentrations in each study region. LF, lumefantrine; DLF, desbutyl-lumefantrine; SD, sulfadoxine; PM, pyrimethamine; MQ, mefloquine; Q, quinine; CQ, chloroquine; AQ, amodiaquine; DAQ, N-desethyl-amodiaquine.

Antimalarial drugs were present in the blood of 20.8% (1330/6391) (95%CI: 20.0–21.6) of individuals in total: 53.4% (710/1330) had LF, 14.2% (189/1330) DLF, 20.5% (272/1330) SD, 26.5% (352/1330) PM, 1.9% (26/1330) Q, 0.6% (8/1330) MQ, 0.2% (3/1330) CQ, 0.1% (2/1330) AQ and 6.5% (86/1330) DAQ. Additionally, 59.5% (792/1330) of individuals had LF and/or DLF (107 individuals had both and 685 had one or the other) and 38.3% (510/1330) SP. When considering parent drug and metabolite or combined treatment as one drug (LF/DLF, AQ/DAQ and SP), 6.6% (88) of the 1330 participants with residual drug concentrations in the blood had more than one antimalarial in the blood: 84 individuals had two different drugs, 2 had three drugs and 2 had five drugs. The proportions of participants with each type of antimalarial in their blood per region are detailed in Fig 1. In Mtwara, the proportion of individuals with LF/DLF in their blood [74.1% (303/409)] was significantly higher than in Mwanza [52.5% (338/644), p<0.001] and Mbeya [54.5% (151/277), p<0.001]. Inversely, the proportion of individuals with SP in their blood was significantly higher in Mwanza [43.6% (281/644), p<0.001] and Mbeya [48.4% (134/277), p<0.001] than in Mtwara [23.2% (95/409)].

### Variables associated with the presence of antimalarials

**Fig 2:**
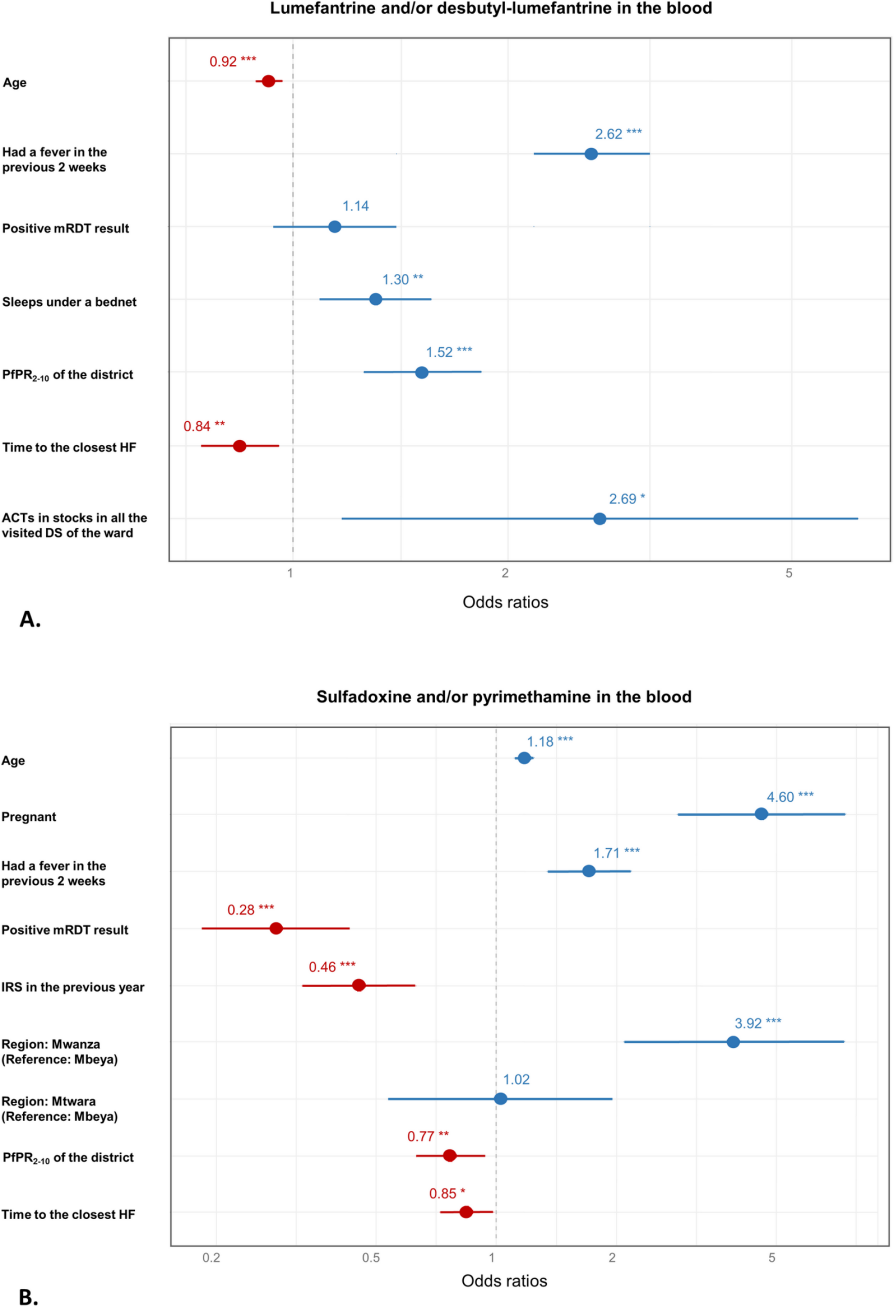
**Multivariate analysis of the determinants for the presence of LF/DLF (A) and SP (B) in the blood of the surveyed individuals.** Lines width corresponds to 95% CI bounds and the number above each line correspond to the association’s OR. * indicate the strength of the significance (levels of significance: * = p<0.05, ** = p<0.01, *** = p<0.001) obtained from the multivariate analysis.

The full list of variables and interaction terms investigated in the multivariate logistic regression model to assess their association with LF/DLF or SP in the blood are summarized in Table 3, while the results of the bivariate and multivariate analysis can be found in Tables 1 and 2. In the multivariate analysis, ignoring interaction terms (Fig 2), an increasing age (OR = 0.9, p<0.001) and living further away from a HF (OR = 0.8, p = 0.007) were associated with lower odds of detecting LF/DLF. Having had a fever in the previous two weeks (OR = 2.6, p<0.001), living in a district with higher malaria prevalence (OR = 1.5, p<0.001), sleeping under a bed net (OR = 1.3, p = 0.004) and living in a ward in which all visited drug stores (DSs) had ACTs in stocks (OR = 2.7, p = 0.020) were associated with higher odds of having DLF/LF in the blood. When considering interactions in the model (S1 File), a statistically significant interaction between age and mRDT result (p<0.001) was noted and showed that increasing age was associated with lower odds of having LF/DLF in the blood only for the individuals tested positive by mRDT. A significant interaction between district parasite prevalence and fever (p<0.001) also showed that the likelihood of having LF/DLF in the blood according to fever was higher at low endemicity than at high endemicity.

**Table 3:** Multivariate logistic regression models used in the present analysis.

	LF/DLF Model	SP Model
**Outcome**	Presence of lumefantrine and/or desbutyl-lumefantrine in the blood	Presence of sulfadoxine and/or pyrimethamine in the blood
**Regions**	All	All
**Number of observations**	6279	6278
**Variables**		
Included	Age; fever in the previous 2 weeks; mRDT result; net use; PfPr of the district; time to the closest HF; ACTs in stocks in DS	Age, pregnant, fever in the previous 2 weeks, mRDT result, IRS in the previous year, region, time to the closest HF
Excluded	Sex; pregnant; IRS in the previous year; area; region; PfPr of the ward; time to the closest DS; ACTs, AM, mRDTs in stocks in HF; AM, mRDTs in stocks in DS	Sex; net use; area; PfPr of the district and of the ward; time to the closest DS; ACTs, AM, mRDTs in stocks in HF; AM, ACTs, mRDTs in stocks in DS
**Nested random effects**	Regions/districts/wards	Regions/districts/wards
**Interaction terms included**	Age*mRDT result; PfPr of the district* fever in the previous 2 weeks	Region* pregnant; Region*time to the closest HF

Concerning the model for SP, being pregnant (OR = 4.6, p<0.001), living in Mwanza (OR = 3.9, p<0.001 compared to Mbeya), having had a fever in the previous two weeks (OR = 1.7, p<0.001) and increasing age (OR = 1.2, p<0.001) were associated with higher odds of having SP in the blood. Inversly, being tested positive by mRDT (OR = 0.3, p<0.001), living in a house that had been sprayed by IRS in the previous year (OR = 0.5, p<0.001), living in a district with higher malaria prevalence (OR = 0.8, p = 0.010) and living further from a HF (OR = 0.8, p = 0.031) were associated with lower odds of having SP in the blood. Considering the interaction between region and pregnancy (p = 0.002) showed that the likelihood of having SP in the blood when pregnant was much higher in Mbeya and Mtwara than in Mwanza (S1 File). The significant interaction between region and time to the closest HF (p = 0.002) revealed that individuals living further from a HF were less likely to have SP in their blood in Mwanza only.

## Discussion

Knowing how antimalarial medication circulates in the population and what the most important drivers of antimalarial consumption are may help to improve treatment appropriateness and prevent the emergence and spread of resistant parasites. To our knowledge, this is the first study assessing drug consumption by means of antimalarial blood concentrations including such a large sample size [19,20,25].

Overall, 20.8% of the study participants had antimalarials in the blood. LF/DLF composed more than half (59.5%) of the drugs detected in our sampled population. Most of the factors significantly associated with the presence of LF/DLF in the blood were expected, including history of fever, which had the highest ORs. Indeed, when fever occurs, antimalarials and antibiotics are commonly used as presumptive treatment [19,24], with or without parasitological confirmation. However, odds of having LF/DLF in the blood according to fever were modulated by district parasite prevalence and were found to be higher at low endemicity. This could be explained by the fact that districts with highest malaria endemicity are also the most rural. Shortage of drugs and long waiting time at HFs due to disease burden, long distance to HFs and inability to pay for health care are factors discouraging people to seek treatment in case of fever and which might not apply in urban districts where inhabitants are wealthier and access to care is less of a concern [11,37,38]. The association between younger age and the presence of LF/DF in the blood adds to previous studies reporting that small children are more likely to be taken to the HF and being treated with ACTs [19,23,25,37,38]. The interaction between age and mRDT result revealed that odds of having LF/DLF in the blood in case of positive mRDT decreased when age increased. This supports previous observations that children are better cared than older people, which is encouraging since children are the most vulnerable population [39]. The effect of the distance to HF and drug stocks in DSs on the presence of LF/DLF confirms that access to treatment (including distance to health care provider and treatment availability) leads to more drug consumption, and hence drug pressure [10,19,40]. In our data, distance to DS did not play a role on pattern of drug use, probably because all households were located within a short radius of DSs. Such leveling effect has previously been reported in the literature [10]. We could not detect an effect of drugs availability in HFs on antimalarial use since all HFs visited during the survey had ACTs in stock. However, the significant effect of stocks of ACTs in DSs shows the importance of these providers, as often reported in the literature [29,41].

SP accounted for 38.3% of all antimalarials detected although it has been shown to have low therapeutic value in Tanzania [4,9], and that SP super-resistant haplotypes are widespread throughout the country [8]. SP had been officially abandoned as first line treatment in 2006 in Tanzania [4] but is still present in HFs for IPTp [4]. Amongst all individuals, 8.0% (510/6391) had SP in the blood. According to 2015–16 DHS surveys [42], 8.6% of women aged between 15–49 years are pregnant on average. Considering that women aged between 15–49 years represent around 25.0% of the total population [43], one would expect around 2.0% of the participants to have SP in their blood, much lower than the proportion measured in our study. As mentioned before, this large discrepancy goes in line with previous studies reporting the common use of SP as self-medication [11,22,44]. Measuring SP in the blood provides interesting insight on how these treatments are used in different regions of the country and by different population groups. For example, in the univariate analysis, female were more likely to have SP in the blood. However, this association was no longer significant when adjusting for pregnancy in the multivariate analysis as previously reported in the literature [19,20,23,28]. Indeed, it was pregnancy that was a strong predictor of the presence of SP in the blood, which reflects adherence to IPTp programs [4]. This association was nevertheless mitigated in Mwanza, the reason possibly being that in Mbeya and Mtwara the use of SP is more restricted to IPTp while in Mwanza, treating malaria with SP is more common practice. This hypothesis is reinforced by the fact that Mwanza was the region with the highest proportion of individuals with SP in the blood, and the only region for which distance to HF had significant influence on the presence of SP in the blood. Age was also significantly associated with the presence of SP in the blood but contrary to DLF/LF, residual levels of SP were more likely to occur in older people, who have more often recourse to self-medication [45]. These results tend to show that compliance to standard malaria treatment recommendations is lower in Mwanza and in adults.

Almost all antimalarials detected in the studied population were part of the recommended treatments in Tanzania, which is encouraging. Beside LF/DLF and SP, which were the most prevalent, 123 individuals (9.3% of all antimalarials detected) were found with quinine, mefloquine, amodiaquine or its metabolite N-desethyl-amodiaquine (which were more prevalent due to amodiaquine’s short half-life of 3.9h when used in ACTs [46]). Quinine is the drug of choice for treatment of severe malaria and second line drug in case of treatment failure with Alu. Amodiaquine and mefloquine can both be found Tanzania in combination with artesunate as a viable option for treating uncomplicated malaria [4]. Three individuals had chloroquine in their blood although it had been banned from the therapeutic armamentarium in 2001.

Previous studies conducted in Tanzania reported that around 2.0% of the fever in low and 8–20% in highly endemic settings ([47–49] Boillat *et al*, unpublished) are due to malaria. In our study, 1021 individuals reported a fever in the previous two weeks (Tables 1 and 2), and 630 of them said they sought care because of their fever [29]. Considering that, on average, 10.0% of fevers are due to malaria according to literature, 63 individuals should have received an antimalarial. This number can be doubled because LF can be detected in DBS samples for up to one month (unpublished data) and history of fever recall was based on a two-week period. These estimations should have resulted in a prevalence of 19.7% (1260/6391) of the total surveyed population (providing all participants who sought care in the previous month were treated) having LF detected in their blood if none had a diagnostic test done and 2.0% (126/6391) if all had been tested and treated upon result. Together with the 2.0% of the population constituted by pregnant women receiving SP through IPTp, we would expect 4.0% of the participants to have an antimalarial in the blood, which is far from what we measured (20.8%). This highlights the urgent need to implement interventions aimed at decreasing drug pressure, notably encourage the use of mRDTs to target treatments to individuals with malaria only.

Our study’s main limitation resides in the fact that we did not collect all possible information on potential factors influencing drug use. Socio-economic determinants, knowledge and attitude on malaria management might have been of interest, although differences in wealth might not have been much different, at least in rural areas.

## Conclusion

Understanding pattern of antimalarial drug use has implication in malaria control (access to care as well as diagnostic and treatment policies implementation) and surveillance (parasite resistance). The most significant predictors identified were encouraging since they implied that people took a drug when they had a fever and that children were treated with standard first line treatment, i.e. artemether-lumefantrine. However, the long-standing belief that access to treatment should be increased might need to be revisited [50], as proven by the number of people having drugs in their blood and the good availability of drugs in the health facilities and drug stores. Efforts need rather to be focused on the appropriate use of these drugs, namely to target antimalarial treatment to those who have malaria, with the appropriate dosage and correct adherence, and to recommend the use first-line efficacious drugs in adults [51]. This study has shown that drug pressure was highest in districts of high transmission and in most accessible locations. This observation calls for interventions to be targeted to such areas since they constitute favorable conditions for development and spread of resistant strains.

## Supporting information

S1 File Logistic regression including interaction terms. (DOCX)

S2 File Survey questionnaires. (PDF)

## Abbreviations

ACTs: Artemisinin-based combination therapies
ADDOs: Accredited Drug Dispensing Outlets
Alu: Artemether-lumefantrine
AQ: Amodiaquine
CQ: Chloroquine
DBS: Dried blood spot
HF: Health facility
IPTp: Intermittent Preventive Treatment of malaria in pregnancy
IRS: Indoor residual spraying
LC-MS/MS: Liquid chromatography coupled to tandem mass-spectrometry
LF: Lumefantrine
LF/DLF: Lumefantrine and/or desbutyl-lumefantrine
LOQ: Limit of quantification
MQ: Mefloquine
mRDTs: malaria rapid diagnostic tests
OR: Odds ratio
PM: Pyrimethamine
Q: Quinine
SD: Sulfadoxine
WHO: World Health Organization
