# Supplementary material for: Predictors of residual antimalarial drugs in the blood in community surveys in Tanzania
Source: PLoS One. 2018 Sep 7;13(9):e0202745. doi: 10.1371/journal.pone.0202745 (PMC6128528; doi:10.1371/journal.pone.0202745)
Supplement: S1 File — (DOCX) [file pone.0202745.s001.docx]

**S1 file. Logistic regression including interaction terms**

S1 Table. Odds ratios of having LF/DLF or SP in the blood estimated by multivariate logistic regression including interaction terms. These tables only present the variables included in the final adjusted models.

| **Participants with LF/DLF in the blood** | | |
| --- | --- | --- |
|  | Multivariate analysis | |
|  | ORs | *p*-value |
| **Variables** |  |  |
| Age | 0.99 | 0.560 |
| Had a fever in the previous 2 weeks | 17.29 | <0.001 |
| mRDT result | 3.65 | <0.001 |
| Net use | 1.31 | 0.003 |
| PfPR_2-10_ of the district | 2.01 | <0.001 |
| Time to the closest HF | 0.84 | 0.006 |
| ACTs in stocks in all the visited DS of the ward | 2.52 | 0.029 |
|  |  |  |
| **Interactions terms** |  |  |
| Age*mRDT result | 0.68 | <0.001 |
| PfPR_2-10_ of the district*fever in the previous 2 weeks | 0.75 | <0.001 |
| **Participants with SP in the blood** | | |
|  | Multivariate analysis | |
|  | ORs | *p*-value |
| **Variables** |  |  |
| Age | 1.18 | <0.001 |
| Pregnant | 12.61 | <0.001 |
| Had a fever in the previous 2 weeks | 1.70 | <0.001 |
| mRDT result | 0.29 | <0.001 |
| IRS in the previous year | 2.17 | <0.001 |
| Region |  |  |
| Mwanza | 11.30 | <0.001 |
| Mtwara | 1.68 | 0.323 |
| PfPR_2-10_ of the district | 0.77 | 0.013 |
| Time to the closest HF | 1.20 | 0.211 |
|  |  |  |
| **Interactions terms** |  |  |
| Mwanza*Pregnant | 0.16 | 0.003 |
| Mtwara*Pregnant | 0.74 | 0.656 |
| Mwanza*Time to the closest HF | 0.57 | 0.002 |
| Mtwara*Time to the closest HF | 0.76 | 0.223 |

When including a statistically significant interaction between age and mRDT result (p<0.001), odds of having LF/DLF in the blood decreased with increasing age, but only for the individuals tested positive (OR=0.67 for those tested positive and OR=0.99 for those tested negative). Participants with a positive mRDT result were more likely to have LF/DLF in the blood (OR=3.6, p<0.001), but ORs decreased when age increased (OR=2.4). After including an interaction between district parasite prevalence and fever (p<0.001), the OR of having LF/DLF in the blood according to fever were higher at low endemicity (OR=8.0) than at high endemicity (OR=6.0). Participants were more likely to have LF/DLF in the blood when living in a district of higher endemicity, especially if they did not have a fever (OR=1.6 for those who did not have a fever and OR=1.2 for those who had a fever).

An interaction between region and pregnancy (p=0.002) showed that pregnant women were more likely to have SP in their blood, especially if they lived in Mbeya and Mtwara region (OR=12.7 in Mbeya, OR=9.5 in Mtwara and OR=1.8 in Mwanza). Odds of having SP in the blood were higher when living in Mwanza, but to a lesser extent for pregnant women (OR=1.8 for pregnant women and OR=11.2 for other participants). An interaction between region and time to the closest HF (p=0.002) revealed that odds of having SP in the blood significantly decreased when living further from a HF, but only for those living in Mwanza (OR=0.7). This association was not significant when living in Mbeya (OR=1.2, p=0.211) or Mtwara (OR=0.9, p=0.223). Furthermore, participants living in Mwanza were more likely to have SP in the blood, especially when living close to a HF (OR=11.2 when living close to a HF and OR=6.4 with increasing distance).
