# Supplementary material for: Predictors of residual antimalarial drugs in the blood in community surveys in Tanzania
Source: PLoS One. 2018 Sep 7;13(9):e0202745. doi: 10.1371/journal.pone.0202745 (PMC6128528; doi:10.1371/journal.pone.0202745)
Supplement: S2 File — (PDF) [file pone.0202745.s002.pdf]

HOUSEHOLD SURVEYS INTERVIEWS

DEMOGRAPHIC FORM

General information:

Staff initials: |__|__|__| House N°: |__|__|

Date of survey (Day/Month/Year): |__|__ / |__|__ / |__|__||__|__|

Region: ....District: ....

Ward:... EA: ....

Altitude: Latitude: Longitude:..

Information about the household:

1. Are you able to interview the household? ❑ Yes ❑No<br>*Note: If the answer is "No", go to question 2.*
2. Why could you not interview the household?<br>❑ No one home ❑ Can't find the location<br>❑ Empty/abandoned house ❑ Refused<br>❑ Other
3. What is the household head name?
4. How long does it take you to to travel from your home to the nearest government health facility by the way you usually travel?<br>❑ Less than 15 min ❑ 2 hours and more<br>❑ From 15-59min ❑ Unknown<br>❑ Between 1 and 2 hours
5. How long does it take you to to travel from your home to the nearest non government health facility (mission or private) by the way you usually travel?<br>❑ Less than 15 min ❑ 2 hours and more<br>❑ From 15-59min ❑ Unknown<br>❑ Between 1 and 2 hours
6. How long does it take you to to travel from your home to the nearest medical shop by the way you usually travel?<br>❑ Less than 15 min ❑ 2 hours and more<br>❑ From 15-59min ❑ Unknown<br>❑ Between 1 and 2 hours

HOUSEHOLD SURVEYS INTERVIEWS

DEMOGRAPHIC FORM

7. At any time in the past 12 months, has anyone sprayed the interior walls of your dwelling against mosquitoes?

❑ Yes ❑ No ❑ Don't know

If yes, how many months ago was the house sprayed? |__|__| months

Go to member list form

HOUSEHOLD SURVEYS INTERVIEWS

MEMBER LIST FORM

General information :

"Now I would like some information about the people who usually live in your household or who are staying with you now. Please give me the names of the persons who usually live in your household and guests of the household who stayed here last night, starting with you (head of the household), and continuing from oldest to youngest. Even if they are not around now but slept here last night I will be back to interview them later."

Information about the household members: *(Answer the following questions by asking the household head)*

Member names:

1.	❑ M	❑ F	Member N°:|__|__|__|__|
2.	❑ M	❑ F	Member N°:|__|__|__|__|
3.	❑ M	❑ F	Member N°:|__|__|__|__|
4.	❑ M	❑ F	Member N°:|__|__|__|__|
5.	❑ M	❑ F	Member N°:|__|__|__|__|
6.	❑ M	❑ F	Member N°:|__|__|__|__|
7.	❑ M	❑ F	Member N°:|__|__|__|__|
8.	❑ M	❑ F	Member N°:|__|__|__|__|

Are there any other persons such as small children or infants that we have not listed?

1.	❑ M	❑ F	Member N°:|__|__|__|__|
2.	❑ M	❑ F	Member N°:|__|__|__|__|
3.	❑ M	❑ F	Member N°:|__|__|__|__|
4.	❑ M	❑ F	Member N°:|__|__|__|__|
5.	❑ M	❑ F	Member N°:|__|__|__|__|
6.	❑ M	❑ F	Member N°:|__|__|__|__|
7.	❑ M	❑ F	Member N°:|__|__|__|__|
8.	❑ M	❑ F	Member N°:|__|__|__|__|

HOUSEHOLD SURVEYS INTERVIEWS

MEMBER LIST FORM

In addition, are there any other people who may not be members of your family, such as domestic servants, friends who usually live here?

1. ❑ M ❑ F Member N°:|__|__|__|__|
2. ❑ M ❑ F Member N°:|__|__|__|__|
3. ❑ M ❑ F Member N°:|__|__|__|__|
4. ❑ M ❑ F Member N°:|__|__|__|__|
5. ❑ M ❑ F Member N°:|__|__|__|__|
6. ❑ M ❑ F Member N°:|__|__|__|__|
7. ❑ M ❑ F Member N°:|__|__|__|__|
8. ❑ M ❑ F Member N°:|__|__|__|__|

Does your household have any mosquito nets that can be used while sleeping? ❑ Yes ❑ No

If yes, how many? |__|__| nets

Go to roster form

HOUSEHOLD SURVEYS INTERVIEWS

ROSTER FORM

General information:

"Now I would like to ask questions related to each members of your Household starting with you. Kindly call them for me when we finish, one at the time. For children, I would like to talk to their caregiver."

Information about the member: *(Answer the following questions by asking the household member)*

1. What is the name of the person you want to interview? *(If you are interviewing caretaker, choose the name of the child here)* .... Member N°:|__|__|__|__|			
2. Does (name) usually live here?	❑ Yes	❑ No	
3. Did (name) stay here last night?	❑ Yes	❑ No	
4. Is it possible to interview him/her (or caretaker)?	❑ Yes	❑ No	
5. If no, why not? ❑ Refuse ❑ Out ❑ Unable to talk ❑ Busy with work ❑ Other :			
6. Is the patient male or female?	❑ M	❑ F	
7. What is the patient's weight in kg?	Kilo |__|__|__|	❑ Don't know	
8. What is the patient's year of birth?	Year |__|__||__|__|	❑ Don't know	
9. When is the patient's birth month?	Month |__|__|	❑ Don't know	
10. When is the patient's birth day?	Day |__|__|	❑ Don't know	
11. Did the patient sleep under a mosquito net last night?	❑ Yes	❑ No	❑ Don't know
12. Did this person have an illness with fever or malaria in the past 14 days? *If the answer is "No", go to question 14, then end questionnaire.*	❑ Yes	❑ No	❑ Don't know
13. Have you taken any antimalarial drug in the last 4 weeks/months?	❑ Yes	❑ No	❑ Don't know

HOUSEHOLD SURVEYS INTERVIEWS

ROSTER FORM

14. If a woman aged between 15-49 years: is she currently pregnant or does she thinks she could be pregnant?

❑ Yes ❑ No ❑ Don't know

What was the last time she had her menstrual period?

❑ Less than one month ago ❑ More than one month ago ❑ Don't know

If the answer is "more than one month ago" or "don't know", please refer that woman to a health facility if she has a RDT positive, and do NOT give her Alu!

---

15. How many days ago did the illness begin? |__|__|

---

16. Are you (is your child) well today? ❑ Yes ❑ No ❑ Don't know

If "Yes", how many days did the illness last? |__|__|

---

17. Did you (or your child) seek any treatment or medicine for this illness? ❑ Yes ❑ No ❑ Don't know

If "Yes", go to question 18.

---

18. Specify the name and place you sought care? If many, record one drug prescribed at a time, choosing amongst the list below:

1. Home
2. Kiosk, stand or general store
3. Duka la dawa baridi
4. ADDO (duka la dawa muhimu)
5. Part I Pharmacy
6. Street doctor
7. Village/community health worker
8. Government health facility
9. Private or mission health facility
10. Traditional practitioner
11. Neighbor or firend
12. Another place
13. Don't know

1st place visited:.................................... 2nd place visited:....................................

3rd place visited:.................................... 4th place visited:....................................

---

19. What is the name of the place you thought care?

1st place visited:.................................... 2nd place visited:....................................

3rd place visited:.................................... 4th place visited:....................................

---

20. How many days after fever began did you first seek care at this source? (0=same day as onset of fever)?

1st place visited: |__|__| ❑ Don't know

2nd place visited: |__|__| ❑ Don't know

3rd place visited: |__|__| ❑ Don't know

4th place visited: |__|__| ❑ Don't know

HOUSEHOLD SURVEYS INTERVIEWS

ROSTER FORM

21. How did you travel to this provider?

1st place visited:	❑ By foot	❑ Bicycle	❑ Motorcycle	❑ Public transport	❑ Other
2nd place visited:	❑ By foot	❑ Bicycle	❑ Motorcycle	❑ Public transport	❑ Other
3rd place visited:	❑ By foot	❑ Bicycle	❑ Motorcycle	❑ Public transport	❑ Other
4th place visited:	❑ By foot	❑ Bicycle	❑ Motorcycle	❑ Public transport	❑ Other

22. How much time did it take to travel to this provider?

1st place visited:	❑ <15 min	❑ 15 min to 1 h	❑ 1h -2h	❑ > 2 hours	❑ Don't know
2nd place visited:	❑ <15 min	❑15 min to 1 h	❑ 1h -2h	❑ > 2 hours	❑ Don't know
3rd place visited:	❑ <15 min	❑ 15 min to 1 h	❑ 1h -2h	❑ > 2 hours	❑ Don't know
4th place visited:	❑ <15 min	❑ 15 min to 1 h	❑ 1h -2h	❑ > 2 hours	❑ Don't know

23. Was a blood test for malaria performed?

1st place visited:	❑ Yes	❑ No	❑ Don't know
2nd place visited:	❑ Yes	❑ No	❑ Don't know
3rd place visited:	❑ Yes	❑ No	❑ Don't know
4th place visited:	❑ Yes	❑ No	❑ Don't know

If "No" or "Don't know", go to question 26

24. Was the test a blood smear or a rapid diagnosis test (RDT)?

1st place visited:	❑ Yes	❑ No	❑ Don't know
2nd place visited:	❑ Yes	❑ No	❑ Don't know
3rd place visited:	❑ Yes	❑ No	❑ Don't know
4th place visited:	❑ Yes	❑ No	❑ Don't know

25. What was the result of the test?

1st place visited:	❑ Positive	❑ Negative	❑ Don't know
2nd place visited:	❑ Positive	❑ Negative	❑ Don't know
3rd place visited:	❑ Positive	❑ Negative	❑ Don't know
4th place visited:	❑ Positive	❑ Negative	❑ Don't know

26. Was a drug prescribed?

1st place visited:	❑ Yes	❑ No	❑ Don't know
2nd place visited:	❑ Yes	❑ No	❑ Don't know
3rd place visited:	❑ Yes	❑ No	❑ Don't know
4th place visited:	❑ Yes	❑ No	❑ Don't know

HOUSEHOLD SURVEYS INTERVIEWS

ROSTER FORM

If "No" or "Don't know", go to question 28

27. Which drug(s) were prescribed? If many, record one drug prescribed at a time, choosing amongst the list below:

1. Artemether-lumefantrine	2. Artesunate-Amodiaquine	3.Other ACT
4. SP/Fansidar	5. Amodiaquine	6.Quinine
7. Mefloquine	8. Artemisinin Monotherapy	9.Amoxicillin
10.Cotrimoxazole	11. Antibiotic	12. Antipyretic
13.Traditional herbs	14. Don't know	15. Other (specify)

(On the line, enter the number corresponding to the drugs like in the list above):

1st place visited: .... 2nd place visited: ....

3rd place visited: .... 4th place visited: ....

28. Were you (or your child) admitted?

1st place visited: ❑ Yes ❑ No ❑ Don't know
2nd place visited: ❑ Yes ❑ No ❑ Don't know
3rd place visited: ❑ Yes ❑ No ❑ Don't know
4th place visited: ❑ Yes ❑ No ❑ Don't know

If "No" or "Don't know", go to question 30

29. If yes, how many days were you admitted?

1st place visited: |__|__| days ❑ Don't know
2nd place visited: |__|__| days ❑ Don't know
3rd place visited: |__|__| days ❑ Don't know
4th place visited: |__|__| days ❑ Don't know

30. Was a drug obtained?

1st place visited: ❑ Yes ❑ No
2nd place visited: ❑ Yes ❑ No
3rd place visited: ❑ Yes ❑ No
4th place visited: ❑ Yes ❑ No

31. If "No", why was the drug not obtained?

1st place visited: ❑ Too expensive ❑ No drug available ❑ Other (specify): ....
2nd place visited: ❑ Too expensive ❑ No drug available ❑ Other (specify): ....
3rd place visited: ❑ Too expensive ❑ No drug available ❑ Other (specify): ....
4th place visited: ❑ Too expensive ❑ No drug available ❑ Other (specify): ....

HOUSEHOLD SURVEYS INTERVIEWS

ROSTER FORM

32. If a drug was obtained, which drug(s) were obtained? If many, record one drug prescribed at a time, choosing amongst the list below:

1. Artemether-Lumefantrine	2. Artesunate-Amodiaquine	3.Other ACT
4. SP/Fansidar	5. Amodiaquine	6.Quinine
7. Mefloquine	8. Artemisinin Monotherapy	9.Amoxicillin
10.Cotrimoxazole	11. Antibiotic	12. Antipyretic
13.Traditional herbs	14. Don't know	15. Other (specify)

(On the line, enter the number corresponding to the drugs like in the list above):

1st place visited:.. 2nd place visited:..

3rd place visited:.. 4th place visited:..

From another place:.. *(Then go to question 33, otherwise go to question 35)*

33. If a drug was obtained from another place, specify the place:

- ❑ Home
- ❑ Kiosk, stand or general store
- ❑ Duka la dawa baridi
- ❑ ADDO (duka la dawa muhimu)
- ❑ Part I Pharmacy
- ❑ Street doctor
- ❑ Village/community health worker
- ❑ Government health facility
- ❑ Private or mission health facility
- ❑ Traditional practitioner
- ❑ Neighbor or friend
- ❑ Another place
- ❑ Don't know

34. Why did you get that drug from this other place?

- ❑ Cheaper
- ❑ Closer
- ❑ It's the place I usually go
- ❑ The drug wasn't available in other sources visited

35. How long after the fever started did you (or your child) first take this drug? *(0=same day)*

1st drug: |__|__| days 2nd drug: |__|__| days

3rd drug: |__|__| days 4th drug: |__|__| days

36. What quantity of the drug were you given in total? (number of tablets/bottles)

1st drug: |__|__|	❑ Don't know	2nd drug: |__|__|	❑ Don't know
3rd drug: |__|__|	❑ Don't know	4th drug: |__|__|	❑ Don't know

37. Was all of this quantity of drug used up in treating this episode of fever?

1st drug	❑ Yes	❑ No	❑ Don't know
2nd drug	❑ Yes	❑ No	❑ Don't know
3rd drug	❑ Yes	❑ No	❑ Don't know
4th drug:	❑ Yes	❑ No	❑ Don't know

38. What quantity of the drug have you taken? (number of tablets/bottles)

1st drug: |__|__|	❑ Don't know	2nd drug: |__|__|	❑ Don't know

HOUSEHOLD SURVEYS INTERVIEWS

ROSTER FORM

3rd drug: |__|__|	❑ Don't know	4th drug: |__|__|	❑ Don't know

39. For how many days did you (or your child) take this antimalarial?

1st drug: |__|__| days	❑ Don't know	2nd drug: |__|__| days	❑ Don't know
3rd drug: |__|__| days	❑ Don't know	4th drug: |__|__| days	❑ Don't know

Go to lab form

HOUSEHOLD SURVEY INTERVIEWS

LAB FORM

Take your lab log book to enter lab results for this person

**1.** Did the person or caretaker agree to take fingerstick for RDT?<br><br>*Note : if the answer is no, end questionnaire.*		❑ Yes	❑ No
**2.** RDT taken?<br><br>*Note : if the answer is no, skip to question 5 otherwise go to question 3*		❑ Yes	❑ No
**3.** RDT Result	❑ Positive	❑ Negative	❑ Not valid
**4.** Do the patient already get appropriate treatment for malaria? ❑ Yes ❑ No<br><br>*Note : if the answer is yes, go to question 6 otherwise go to question 5*			
**5.** Did you give Alu to treat malaria? ❑ Yes ❑ No<br><br>*If the patient is a woman and think she is pregnant, do NOT give Alu, but refer her to a health facility!*			
**6.** Did this person need to be directed to a health facility today? ❑ Yes ❑ No<br><br>*Please make sure that you haven't missed one severe symptom or complain from the patient. Take time to ask him about that matter.*			

End questionnaire

HOUSEHOLD SURVEYS INTERVIEWS

DEMOGRAPHIC FORM

Taarifa za jumla:

Herufi za mwanzo za majina ya mfanyakazi: |__|__|__| Nyumba N°: |__|__|

Tarehe ya utafiti (Siku/Mwezi/Mwaka): |__|__ / |__|__ / |__|__||__|__|

Mkoa: .... Wilaya: ....

Kata: .... EA: ....

Altitudo: Latitudo: Longitudo: ....

Taarifa kuhusu Kaya:

1. Je, unaweza kuhoji kaya hii?? ❑ Ndiyo ❑Hapana

   Kumbuka: kama jibu ni "Hapana" nenda swali namba 2.

2. Kwa nini hukuweza kuhoji kaya hii??
   ❑ Hakuna mtu nyumbani ❑ Siwezi kupata eneo ❑ Tupu/nyumba imetelekezwa ❑ Kakataa
   ❑ Nyingine

3. Jina la Mkuu wa Kaya:

4. Je, huwa unatumia muda gani kufika kwenye hospitali ya kiserikali iliyo karibu na kaya yako?
   ❑ Chini ya dakika 15 ❑ Saa 2 au zaidi
   ❑ Dakika 15 hadi 59 ❑ Haijulikani
   ❑ Kati ya saa 1 hadi saa 1 dakika 59

5. Je, huwa unatumia muda gani kufika kwenye hospitali isiyo ya kiserikali(Misheni au Binafsi) iliyo karibu na kaya yako?
   ❑ Chini ya dakika 15 ❑ Saa 2 au zaidi
   ❑ Dakika 15 hadi 59 ❑ Haijulikani
   ❑ Kati ya saa 1 hadi saa 1 dakika 59

6. Je, inakuchukua muda gani kwenda kwenye duka la dawa lililo karibu na nyumbani kwako?
   ❑ Chini ya dakika 15 ❑ Saa 2 au zaidi
   ❑ Dakika 15 hadi 59 ❑ Haijulikani
   ❑ Kati ya saa 1 hadi saa 1 dakika 59

7. Je, katika kipindi cha miezi 12 iliyopita, kuna mtu yoyote aliyepuliza dawa ya kuua mbu kwenye kuta za ndani za sehemu unayokaa?
   ❑ Ndiyo ❑ Hapana ❑ Sijui

HOUSEHOLD SURVEYS INTERVIEWS

DEMOGRAPHIC FORM

Kama ndiyo, je, ni miezi mingapi imepita tangu hiyo nyumba ilipopuliziwa dawa?? |__|__| miezi

"Nenda kwenye fomu ya mahojiano ya mgonjwa"

HOUSEHOLD SURVEYS INTERVIEWS

FOMU YA IDADI YA WANAKAYA

Taarifa za jumla:

"Sasa ningependa kupata taarifa kuhusu watu ambao wanaishi katika kaya yako siku zote au wanaokaa hapa kwa sasa. Tafadhali nitajie majina ya watu wanaoishi katika kaya hii siku zote na wageni waliokaa hapa usiku wa kuamkia leo, kwa kuanzia na jina la mkuu wa kaya, na kuendelea na majina ya wakubwa hadi wadogo.hata kama wametoka kwa muda huu ninaweza kurudi kuwafanyia usahili baadae."

Taarifa kuhusu watu waishio katika kaya : (Jibu maswali yafuatayo kwa kumuuliza mkuu wa kaya)

Majina ya wanakaya:

1. ❑ Me ❑ Ke Nyumba N°:|__|__|__|__|
2. ❑ Me ❑ Ke Nyumba N°:|__|__|__|__|
3. ❑ Me ❑ Ke Nyumba N°:|__|__|__|__|
4. ❑ Me ❑ Ke Nyumba N°:|__|__|__|__|
5. ❑ Me ❑ Ke Nyumba N°:|__|__|__|__|
6. ❑ Me ❑ Ke Nyumba N°:|__|__|__|__|
7. ❑ Me ❑ Ke Nyumba N°:|__|__|__|__|
8. ❑ Me ❑ Ke Nyumba N°:|__|__|__|__|

Je, kuna watu wengine kama vile watoto wadogo au wachanga ambao hawajaorodheshwa?

1. ❑ Me ❑ Ke Nyumba N°:|__|__|__|__|
2. ❑ Me ❑ Ke Nyumba N°:|__|__|__|__|
3. ❑ Me ❑ Ke Nyumba N°:|__|__|__|__|
4. ❑ Me ❑ Ke Nyumba N°:|__|__|__|__|
5. ❑ Me ❑ Ke Nyumba N°:|__|__|__|__|
6. ❑ Me ❑ Ke Nyumba N°:|__|__|__|__|
7. ❑ Me ❑ Ke Nyumba N°:|__|__|__|__|
8. ❑ Me ❑ Ke Nyumba N°:|__|__|__|__|

HOUSEHOLD SURVEYS INTERVIEWS

FOMU YA IDADI YA WANAKAYA

Je, kuna wageni au watembezi wa muda mfupi wanaokaa hapa, au kuna mtu mwingine yeyote aliyelala hapa usiku wa kuamkia leo, ambae hajaorodheshwa?

1.	❑ Me	❑ Ke	Nyumba N°:|__|__|__|__|
2.	❑ Me	❑ Ke	Nyumba N°:|__|__|__|__|
3.	❑ Me	❑ Ke	Nyumba N°:|__|__|__|__|
4.	❑ Me	❑ Ke	Nyumba N°:|__|__|__|__|
5.	❑ Me	❑ Ke	Nyumba N°:|__|__|__|__|
6.	❑ Me	❑ Ke	Nyumba N°:|__|__|__|__|
7.	❑ Me	❑ Ke	Nyumba N°:|__|__|__|__|
8.	❑ Me	❑ Ke	Member N°:|__|__|__|__|

Je, kaya yako ina vyandarua vyovyote vinavyoweza kutumika wakati wa kulala? ❑ Ndiyo ❑ Hapana

nenda fomu ya orodha

HOUSEHOLD SURVEYS INTERVIEWS

ROSTER FORM

Taarifa za jumla:

"Sasa ningependa kufanya mahojiano na wakazi wa kaya yako kwa kuanzia na wewe, tafadhari naomba uniitie mkazi mwingine nikimalizana nawe.Kwa watoto nitapenda kuongea na walezi wao."

Taarifa kuhusu mwana kaya : (Jibu maswali yafuatayo kwa kumuuliza mwana kaya**)**

1. Jina la mtu unayetaka kumfanyia mahojiano? (*Kama unamuhoji mlezi/Mwangalizi , andika jina na mtoto hapa*)<br>--<br>Nyumba N°:|__|__|__|__|			
2. Je, (JINA) kwa kawaida unaishi hapa?	❑ Ndiyo	❑ Hapana	
3. Je, (JINA) ulilala hapa usiku wa kuamkia leo?	❑ Ndiyo	❑ Hapana	
4. Ninaweza kufanya mahojiano na mtu huyu au mlezi?	❑ Ndiyo	❑ Hapana	
5. Kama siwezi kwanini?<br>❑ Amekataa ❑ Hayupo nyumbani ❑ Hawezi kuongea ❑ Hana muda(Busy)<br>❑ Nyingine : --<br>*Kasha maliza mahojiano na nenda kwa mtu anayefuata.*			
6. Je mgonjwa ni mwanaume au mwanamke?	❑ Me	❑ Ke	
7. Je,mgonjwa ana kilo ngapi?	Kilo |__|__|__|		❑ Sijui
8. Mwaka wa kuzaliwa wa mgonjwa?	Mwaka |__|__||__|__|		❑ Sijui
9. Mwezi wa kuzaliwa wa mgonjwa?	Mwezi |__|__|		❑ Sijui
10. Siku ya kuzaliwa ya mgonjwaa?	Siku |__|__|		❑ Sijui
11. Je mgonjwa alilala ndani ya chandarua usiku uliopita?	❑ Ndiyo	❑ Hapana	❑ Sijui
12. Je mtu huyu ameugua homa au malaria katika siku 14 zilizopita?<br>*Kama jibu ni "Ndiyo" nenda Swali la 14, kisha maliza dodoso.*	❑ Ndiyo	❑ Hapana	❑ Sijui

HOUSEHOLD SURVEYS INTERVIEWS

ROSTER FORM

13. Je umeshatumia dawa yoyote ya malaria ndani ya wiki 4/mwezi uliyopita? ❑ Ndiyo ❑ Hapana ❑ Sijui

14. Kama mwanamke ana umri kati ya miaka 15-49: Je ni mjamzito kwa sasa au anafikiri anaweza kuwa mjamzito?

❑ Ndiyo ❑ Hapana ❑ Sijui

Je ni lini mara ya mwisho alipata hedhi?

❑ Chini ya mwezi mmoja ❑ Zaidi ya mwezi mmoja uliopita ❑ Sijui

Kama jibu ni "zaidi ya mwezi mmoja uliopita" au "Sijui", tafadhali mpe rufaa mwanamke huyo kwenda kituo cha tiba kama ana kipimo chanya cha RDT na USIMPATIE Alu!

15. Je, homa ilianza siku ngapi zilizopita? |__|__|

16. Je, unajisikia (mtoto wako anajisikia) vizuri leo? ❑ Ndiyo ❑ Hapana ❑ Sijui

Kama "Ndiyo" ni kwa siku ngapi ugonjwa ulidumu?|__|__|

17. Je wewe (au mwanao) umepata huduma au tiba kwa ugonjwa huu (ikihusu nyumbani pia)?

❑ Ndiyo ❑ Hapana ❑ Sijui

Kama "Ndiyo" nenda swali la 18.

18. Taja jina na mahali, ulikopata huduma? Kama nyingi, rekodi dawa moja iliyotumika kwa wakati, chagua miongoni mwa orodha ya dawa zifuatazo****:

1. Nyumbani	2. Kioski, stendi au duka la kawaida	3. Duka la dawa baridi
4. ADDO (duka la dawa muhimu)	5. Famasi kubwa	6. Daktari wa mtaani
7. Mhudumu wa afya wa kijiji/jamii	8. Kituo cha tiba cha serikali	9. Kituo cha tiba cha misheni au binafsi
10. Mganga wa jadi	11. Jirani au rafiki	12. Sehemu nyingine
13. Sijui		

Sehemu ya kwanza iliyotembelewa:________ Sehemu ya pili iliyotembelewa:________
Sehemu ya tatu iliyotembelewa:________ Sehemu ya nne iliyotembelewa:________

19. Jina la sehemu unapofuata huduma:

Sehemu ya kwanza iliyotembelewa:________ Sehemu ya pili iliyotembelewa:________
Sehemu ya tatu iliyotembelewa:________ Sehemu ya nne iliyotembelewa:________

HOUSEHOLD SURVEYS INTERVIEWS

ROSTER FORM

20. Je, zilipita siku ngapi tangu ulipougua hadi ulipotafuta huduma sehemu hii? (0=siku ya kwanza nipojisikia naumwa)?

Sehemu ya kwanza iliyotembelewa:	|__|__|	❑ Sijui
Sehemu ya pili iliyotembelewa:	|__|__|	❑ Sijui
Sehemu ya tatu iliyotembelewa:	|__|__|	❑ Sijui
Sehemu ya nne iliyotembelewa:	|__|__|	❑ Sijui

21. Je ulitumia usafiri gani kufika kwa mtoa huduma?

Sehemu ya kwanza iliyotembelewa:	❑ Kwa miguu	❑ Baisikeli	❑ Pikipiki	❑ Gari la abiria	❑ Nyingine
Sehemu ya pili iliyotembelewa:	❑ Kwa miguu	❑ Baisikeli	❑ Pikipiki	❑ Gari la abiria	❑ Nyingine
Sehemu ya tatu iliyotembelewa:	❑ Kwa miguu	❑ Baisikeli	❑ Pikipiki	❑ Gari la abiria	❑ Nyingine
Sehemu ya nne iliyotembelewa:	❑ Kwa miguu	❑ Baisikeli	❑ Pikipiki	❑ Gari la abiria	❑ Nyingine

22. Je, ulichukua muda gani kufika kwa mtoa huduma huyo?

Sehemu ya kwanza iliyotembelewa:	❑ < dakika 15	❑ dakika 15 hadi saa 1	❑ saa 1 hadi saa 2	❑ > saa 2	❑ Sijui
Sehemu ya pili iliyotembelewa:	❑ < dakika 15	❑ dakika 15 hadi saa 1	❑ saa 1 hadi saa 2	❑ > saa 2	❑ Sijui
Sehemu ya tatu iliyotembelewa:	❑ < dakika 15	❑ dakika 15 hadi saa 1	❑ saa 1 hadi saa 2	❑ > saa 2	❑ Sijui
Sehemu ya nne iliyotembelewa:	❑ < dakika 15	❑ dakika 15 hadi saa 1	❑ saa 1 hadi saa 2	❑ > saa 2	❑ Sijui

23. Je, ulipimwa damu ya malaria?

Sehemu ya kwanza iliyotembelewa:	❑ Ndiyo	❑ Hapana	❑ Sijui
Sehemu ya pili iliyotembelewa:	❑ Ndiyo	❑ Hapana	❑ Sijui
Sehemu ya tatu iliyotembelewa:	❑ Ndiyo	❑ Hapana	❑ Sijui
Sehemu ya nne iliyotembelewa:	❑ Ndiyo	❑ Hapana	❑ Sijui

Kama "Hapana" au "Sijui", nenda swali la 26.

24. Je, damu iliwekwa kwenye kipimo cha malaria cha darubini au RDT?

Sehemu ya kwanza iliyotembelewa:	❑ Ndiyo	❑ Hapana	❑ Sijui
Sehemu ya pili iliyotembelewa:	❑ Ndiyo	❑ Hapana	❑ Sijui
Sehemu ya tatu iliyotembelewa:	❑ Ndiyo	❑ Hapana	❑ Sijui
Sehemu ya nne iliyotembelewa:	❑ Ndiyo	❑ Hapana	❑ Sijui

25. Majibu yalikuwaje?

Sehemu ya kwanza iliyotembelewa:	❑ Chanya (ana malaria)	❑ Hasi (hana malaria)	❑ Sio halisi
Sehemu ya pili iliyotembelewa:	❑ Chanya (ana malaria)	❑ Hasi (hana malaria)	❑ Sio halisi

HOUSEHOLD SURVEYS INTERVIEWS

ROSTER FORM

Sehemu ya tatu iliyotembelewa: ❑ Chanya (ana malaria) ❑ Hasi (hana malaria) ❑ Sio halisi
Sehemu ya nne iliyotembelewa: ❑ Chanya (ana malaria) ❑ Hasi (hana malaria) ❑ Sio halisi

26. Je, kuna dawa yeyote imeandikwa kwenye kadi kwamba anapaswa kupatiwa?

Sehemu ya kwanza iliyotembelewa: ❑ Ndiyo ❑ Hapana ❑ Sijui
Sehemu ya pili iliyotembelewa: ❑ Ndiyo ❑ Hapana ❑ Sijui
Sehemu ya tatu iliyotembelewa: ❑ Ndiyo ❑ Hapana ❑ Sijui
Sehemu ya nne iliyotembelewa: ❑ Ndiyo ❑ Hapana ❑ Sijui

Kama "Hapana" au "Sijui", nenda swali la 28.

27. Je ni dawa (madawa) gani yalitumika? Kama nyingi, rekodi dawa moja iliyotumika kwa wakati, chagua miongoni mwa orodha ya dawa zifuatazo:

1. Artemether-lumefantrine
2. Artesunate-Amodiaquine
3. Dawa zinginezo za mchanganyiko wa artemisin
4. SP/Fansidar
5. Amodiaquine
6.Quinine
7. Mefloquine
8. Artemisinin Monotherapy
9.Amoxicillin
10.Cotrimoxazole
11. Antibiotic
12. Antipyretic
13. Miti shamba
14. Sijui
15. Nyingine (Taja)

(Katika mstari, jaza namba inayohusianana na madawa kama yalivyo katika orodha hapo juu):

Sehemu ya kwanza iliyotembelewa:.................................... Sehemu ya pili iliyotembelewa:....................................
Sehemu ya tatu iliyotembelewa:.................................... Sehemu ya nne iliyotembelewa:....................................

28. Je, ulilazwa (au mtoto wako alilazwa) kwa mtoa huduma huyo?

Sehemu ya kwanza iliyotembelewa: ❑ Ndiyo ❑ Hapana ❑ Sijui
Sehemu ya pili iliyotembelewa: ❑ Ndiyo ❑ Hapana ❑ Sijui
Sehemu ya tatu iliyotembelewa: ❑ Ndiyo ❑ Hapana ❑ Sijui
Sehemu ya nne iliyotembelewa: ❑ Ndiyo ❑ Hapana ❑ Sijui

Kama "Hapana" au "Sijui", nenda swali la 28.

29. Kama ndiyo, ulilazwa (au mtoto wako alilazwa) kwa siku ngapi?

Sehemu ya kwanza iliyotembelewa: |__|__| Siku ❑ Sijui
Sehemu ya pili iliyotembelewa: |__|__| Siku ❑ Sijui
Sehemu ya tatu iliyotembelewa: |__|__| Siku ❑ Sijui
Sehemu ya nne iliyotembelewa: |__|__| Siku ❑ Sijui

30. Je, ulipatiwa dawa?

Sehemu ya kwanza iliyotembelewa: ❑ Ndiyo ❑ Hapana ❑ Sijui

HOUSEHOLD SURVEYS INTERVIEWS

ROSTER FORM

Sehemu ya pili iliyotembelewa:	❑ Ndiyo	❑ Hapana	❑ Sijui
Sehemu ya tatu iliyotembelewa:	❑ Ndiyo	❑ Hapana	❑ Sijui
Sehemu ya nne iliyotembelewa:	❑ Ndiyo	❑ Hapana	❑ Sijui

31. Kama "hapana", kwanini dawa haikupatikana?

1st place visited:	❑ Gharama kubwa	❑ Dawa haikupatikana dukani	❑ Nyingine (Taja):
2nd place visited:	❑ Gharama kubwa	❑ Dawa haikupatikana dukani	❑ Nyingine (Taja):
3rd place visited:	❑ Gharama kubwa	❑ Dawa haikupatikana dukani	❑ Nyingine (Taja):
4th place visited:	❑ Gharama kubwa	❑ Dawa haikupatikana dukani	❑ Nyingine (Taja):

32. Kama dawa ilipatikana, ni dawa (madawa) yapi yalipatikana? Kama nyingi, rekodi dawa moja iliyotumika kwa wakati, chagua miongoni mwa orodha ya dawa zifuatazo:

1. Artemether-lumefantrine	2. Artesunate-Amodiaquine	3. Dawa zinginezo za mchanganyiko wa artemisin
4. SP/Fansidar	5. Amodiaquine	6.Quinine
7. Mefloquine	8. Artemisinin Monotherapy	9. Amoxicillin
10. Cotrimoxazole	11. Antibiotic	12. Antipyretic
13. Miti shamba	14. Sijui	15. Nyingine (Taja)

(Katika mstari, jaza namba inayohusianana na madawa kama yalivyo katika orodha hapo juu):

Sehemu ya kwanza iliyotembelewa:........................ Sehemu ya pili iliyotembelewa:........................

Sehemu ya tatu iliyotembelewa:........................ Sehemu ya nne iliyotembelewa:........................

Kutoka sehemu nyingine:........................ *(Kisha nenda swali 33, vinginevyo nenda swali namba 35)*

33. Kama dawa ilipatikana kutoka sehemu nyingine , taja sehemu hiyo:

1. Nyumbani	2. Kioski, stendi au duka la kawaida	3. Duka la dawa baridi
4. ADDO (duka la dawa muhimu)	5. Famasi kubwa	6. Daktari wa mtaani
7. Mhudumu wa afya wa kijiji/jamii	8. Kituo cha tiba cha serikali	9. Kituo cha tiba cha misheni au binafsi
10. Mganga wa jadi	11. Jirani au rafiki	12. Sehemu nyingine
13. Sijui		

34. Kwa nini umeenda kuchukuwa dawa sehemu husika uliyotaja?

❑ Bei nafuu ❑ Ukaribu wa duka

❑ Ni sehemu ninayozoea kwenda ❑ Dawa haikupatikana sehemu nyingine nilizotembelea

35. Ni muda gani baada ya homa kuanza wewe au (mtoto wako) alikunywa dawa mara ya kwanza? *(0=siku hiyo hiyo)*

Dawa ya kwanza:	|__|__| siku	Dawa ya pili:	|__|__| siku
Dawa ya tatu:	|__|__| siku	Dawa ya nne:	|__|__| siku

36. Je, ulipewa dawa kiasi gani kwa ujumla? (idadi ya vidonge/ chupa)

Dawa ya kwanza: |__|__| ❑ Sijui Dawa ya pili: |__|__| ❑ Sijui

HOUSEHOLD SURVEYS INTERVIEWS

ROSTER FORM

Dawa ya tatu: |__|__| ❑ Sijui Dawa ya nne: |__|__| ❑ Sijui

37. Je, kiasi chote hiki cha dawa kilitumika kutibu ugonjwa huu/huo?

Dawa ya kwanza:	❑ Ndiyo	❑ Hapana	❑ Sijui
Dawa ya pili:	❑ Ndiyo	❑ Hapana	❑ Sijui
Dawa ya tatu:	❑ Ndiyo	❑ Hapana	❑ Sijui
Dawa ya nne:	❑ Ndiyo	❑ Hapana	❑ Sijui

38. Je ni kiasi gani cha dawa ulichotumia au mtoto wako alichotumia? (idadi ya vidonge/ chupa)

Dawa ya kwanza:	|__|__|	❑ Sijui	Dawa ya pili:	|__|__|	❑ Sijui
Dawa ya tatu:	|__|__|	❑ Sijui	Dawa ya nne:	|__|__|	❑ Sijui

39. Je, umemeza (mtoto wako alimeza)dawa hii ya malaria kwa muda gani?

Dawa ya kwanza:	|__|__|	❑ Sijui	Dawa ya pili:	|__|__|	❑ Sijui
Dawa ya tatu:	|__|__|	❑ Sijui	Dawa ya nne:	|__|__|	❑ Sijui

Nenda kwenye fomu ya maabara

HOUSEHOLD SURVEY INTERVIEWS

FOMU YA MAABARA YA MGONJWA

Chukua kitabu cha taarifa za maabara na ingiza matokeo ya vipimo vya mtu huyu.

**1.** Je, (Jina) au mlezi amekubali kuchukuliwa damu kidoleni ili kupimwa damu ya kipimo cha haraka cha malaria (RDT), filter paper na himoglobini? ❑ Ndiyo ❑ Hapana<br>*Kumbuka: kama jibu ni hapana maliza mahojiano.*
**2.** RDT imechukuliwa? ❑ Ndiyo ❑ Hapana<br>*Kumbuka: kama jibu ni hapana nenda hadi swali la tano vinginevyo nenda swali la 3.*
**3.** Majibu ya RDT: ❑ Chanya (ana malaria) ❑ Hasi (hana malaria) ❑ Sio halisi
**4.** Je mgonjwa tayari amepata matibabu sahihi ya malaria? ❑ Ndiyo ❑ Hapana<br>*Kumbuka: Kama jibu ni ndiyo nenda swali namba 6 vinginevyo nenda swali la 5*
**5.** Umepatia ALU kutibu malaria? ❑ Ndiyo ❑ Hapana<br>*Kama mgonjwa ni mwanamke na unadhani ni mjamzito , USIMPATIE Alu , lakini mpe rufaa kwenye kituo cha tiba.*
**6.** Je mtu huyu aliitajika kurudishwa katika kituo hiki cha afya leo? ❑ Ndiyo ❑ Hapana

Mwisho wa dodoso

HEALTH FACILITY SURVEY – HF STOCKS

STOCKS FORM

General information :

Staff initials: |__|__|__|

Date of survey (Day/Month/Year): |__|__ / |__|__ / |__|__||__|__|

Region: ....District: ....

Ward... Health Facility Name ....

Altitude: Latitude: Longitude:..

Type of health facility: ❑ Hospital ❑ Health centre ❑ Dispensary

Is it a government health facility? ❑ Yes ❑ No ❑ Don't know

EQUIPMENT AND SUPPLIES FOR DIAGNOSTIC TESTING

Which of the following were observed at the health facility in or near at least one consultation room? (make sure you have seen!!)

1. Functional microscope (ask lab worker if it is functional)	❑ Yes ❑ No ❑ Don't know
3. How many glasses for malaria blood smears?	.... ❑ More than 50
5. How many mRDTs in stock today (not expired)?<br>7. Has it been a stockout of RDTs during the last month?<br>8. If yes, for how many days did the stockout last? *If the RDTs were out of stocks more than once in the same month, write the sum of the days.*	.... ❑ More than 50<br>❑ Yes ❑ No ❑ Don't know<br>............... days

STOCKS OF DRUGS

9. How many treatments of **Artemether-Lumefantrine** for **5-14 kg** (not expired) in stock today?<br>11. Has it been a stockout of **Artemether-Lumefantrine** for **5-14 kg** during the last month?<br>12. If yes, for how many days did the stockout last?	.... ❑ More than 50<br>❑ Yes ❑ No ❑ Don't know<br>............... days
13. How many treatments of **Artemether-Lumefantrine** for **15-24 kg** (not expired) in stock today?<br>15. Has it been a stockout of **Artemether-Lumefantrine** for **15-24 kg** during the last month?<br>16. If yes, for how many days did the stockout last?	.... ❑ More than 50<br>❑ Yes ❑ No ❑ Don't know<br>............... days
17. How many treatments of **Artemether-Lumefantrine** for **25-34 kg** (not expired) in stock today?<br>19. Has it been a stockout of **Artemether-Lumefantrine** for **25-34 kg** during the last month?<br>20. If yes, for how many days did the stockout last?	.... ❑ More than 50<br>❑ Yes ❑ No ❑ Don't know<br>............... days
21. How many treatments of **Artemether-Lumefantrine** for **>35kg kg** (not expired) in stock today?	.... ❑ More than 50

HEALTH FACILITY SURVEY – HF STOCKS

STOCKS FORM

23. Has it been a stockout of **Artemether-Lumefantrine** for >**35kg kg** during the last month?<br>24. If yes, for how many days did the stockout last?	❑ Yes ❑ No ❑ Don't know<br>............... days
25. How many injections of **Artemether IM** (not expired) in stock today?<br>27. Has it been a stockout of **Artemether IM** during the last month?<br>28. If yes, for how many days did the stockout last?	.... ❑ More than 50<br>❑ Yes ❑ No ❑ Don't know<br>............... days
29. How many injections of **Artesunate IV** (not expired) in stock today?<br>31. Has it been a stockout of **Artesunate IV** during the last month?<br>32. If yes, for how many days did the stockout last?	.... ❑ More than 50<br>❑ Yes ❑ No ❑ Don't know<br>............... days
33. How many treatments of **Artesunate suppositories** (not expired) in stock today?<br>35. Has it been a stockout of **Artesunate suppositories** during the last month?<br>36. If yes, for how many days did the stockout last?	.... ❑ More than 50<br>❑ Yes ❑ No ❑ Don't know<br>............... days
37. How many treatments of **Artesunate combinations** (not expired) in stock today?<br>39. Has it been a stockout of **Artesunate combinations** during the last month?<br>40. If yes, for how many days did the stockout last?	.... ❑ More than 50<br>❑ Yes ❑ No ❑ Don't know<br>............... days
41. How many treatments of **Dihydroartemisinin** (not expired) in stock today?<br>43. Has it been a stockout of **Dihydroartemisinin** during the last month?<br>44. If yes, for how many days did the stockout last?	.... ❑ More than 50<br>❑ Yes ❑ No ❑ Don't know<br>............... days
45. How many injections of **Artemisinin IV** (not expired) in stock today?<br>47. Has it been a stockout of **Artemisinin IV** during the last month?<br>48. If yes, for how many days did the stockout last?	.... ❑ More than 50<br>❑ Yes ❑ No ❑ Don't know<br>............... days
49. How many treatments of **Artemisinin combinations** (not expired) in stock today?<br>51. Has it been a stockout of **Artemisinin combinations** during the last month?<br>52. If yes, for how many days did the stockout last?	.... ❑ More than 50<br>❑ Yes ❑ No ❑ Don't know<br>............... days
53. How many treatments of **other ACT** (not expired) in stock today?<br>55. Has it been a stockout of **other ACT** during the last month?<br>56. If yes, for how many days did the stockout last?	.... ❑ More than 50<br>❑ Yes ❑ No ❑ Don't know<br>............... days
57. How many treatments of **Amodiaquine** (not expired) in stock today?<br>59. Has it been a stockout of **Amodiaquine** during the last month?<br>60. If yes, for how many days did the stockout last?	.... ❑ More than 50<br>❑ Yes ❑ No ❑ Don't know<br>............... days
61. How many treatments of **Sulfadoxine-Pyrimethamine** (not expired) in stock today?<br>63. Has it been a stockout of **Sulfadoxine-Pyrimethamine** during the last month?<br>64. If yes, for how many days did the stockout last?	.... ❑ More than 50<br>❑ Yes ❑ No ❑ Don't know<br>............... days
65. How many treatments of **Mefloquine** (not expired) in stock today?<br>67. Has it been a stockout of **Mefloquine** during the last month?	.... ❑ More than 50<br>❑ Yes ❑ No ❑ Don't know

HEALTH FACILITY SURVEY – HF STOCKS

STOCKS FORM

68. If yes, for how many days did the stockout last?	.... days
69. How many treatments of **Quinine** (not expired) in stock today?	.... ❑ More than 50
71. Has it been a stockout of **Quinine** during the last month?	❑ Yes ❑ No ❑ Don't know
72. If yes, for how many days did the stockout last?	.... days
73. How many injections of **Quinine IV** (not expired) in stock today?	.... ❑ More than 50
75. Has it been a stockout of injections of **Quinine IV** during the last month?	❑ Yes ❑ No ❑ Don't know
76. If yes, for how many days did the stockout last?	.... days
77. How many treatments of **Atovaquone-Proguanil** (not expired) in stock today?	.... ❑ More than 50
79. Has it been a stockout of **Atovaquone-Proguanil** during the last month?	❑ Yes ❑ No ❑ Don't know
80. If yes, for how many days did the stockout last?	.... days
81. Is there any other antimalarial that has not been listed?	❑ Yes ❑ No ❑ Don't know
82. If yes, what is the name of the antimalarial? *(write the brand name)*	Name: ....
83. How many treatments of this antimalarial (not expired) in stock today?	.... ❑ More than 50
85. Has it been a stockout of this antimalarial during the last month?	❑ Yes ❑ No ❑ Don't know
86. If yes, for how many days did the stockout last?	.... days
81(b). Is there any other antimalarial that has not been listed?	❑ Yes ❑ No ❑ Don't know
82(b). If yes, what is the name of the antimalarial? *(write the brand name)*	Name: ....
83(b). How many treatments of this antimalarial (not expired) in stock today?	.... ❑ More than 50
85(b). Has it been a stockout of this antimalarial during the last month?	❑ Yes ❑ No ❑ Don't know
86(b). If yes, for how many days did the stockout last?	.... days
88. How many treatments of **Amoxicillin in blister** (not expired) in stock today?	.... ❑ More than 50
90. Is there **Amoxicillin** in another form that in blister in stock today?	❑ Yes ❑ No❑ Don't know
91. If yes, please specify the number and the form (example: 2 boxes of 1000 tablets)	....
92. Has it been a stockout of **Amoxicillin** during the last month?	❑ Yes ❑ No❑ Don't know
93. If yes, for how many days did the stockout last?	.... days
95. How many treatments of **Cotrimoxazole in blister** (not expired) in stock today?	.... ❑ More than 50
97. Is there **Cotrimoxazole** in another form that in blister in stock today?	❑ Yes ❑ No❑ Don't know
98. If yes, please specify the number and the form (example: 2 boxes of 1000 tablets)	....
99. Has it been a stockout of **Cotrimoxazole** during the last month?	❑ Yes ❑ No❑ Don't know
100. If yes, for how many days did the stockout last?	.... days
102. How many treatments of **Doxycycline** (not expired) in stock today?	.... ❑ More than 50
104. Has it been a stockout of **Doxycycline** during the last month?	❑ Yes ❑ No ❑ Don't know
105. If yes, for how many days did the stockout last?	.... days
106. How many treatments of **Cloxacilline** (not expired) in stock today?	.... ❑ More than 50

HEALTH FACILITY SURVEY – HF STOCKS

STOCKS FORM

108. Has it been a stockout of **Cloxacilline** during the last month?	❑ Yes ❑ No ❑ Don't know
109. If yes, for how many days did the stockout last?	---------------- days

HEALTH FACILITY SURVEY – HF STOCKS

REGISTER BOOK FORM

General information:

Staff initials: |__|__|__|

Date of survey (Day/Month/Year): |__|__ / |__|__ / |__|__||__|__|

Region: .... District: ....

Ward .... Health Facility Name...

Altitude: Latitude: Longitude: ....

Type of health facility: ❑ Hospital ❑ Health centre ❑ Dispensary

Is it a government health facility? ❑ Yes ❑ No ❑ Don't know

CLINICIAN REGISTER BOOK

**Patients**	
1. Number of patients registered at the facility?	
**Visits**	
2. How many visits have occured during the last two weeks?	
**Malaria tests**	
3. How many patients were tested for malaria (mRDTs or microscopy) during the last two weeks?	
**Positive tests**	
4. How many patients tested for malaria had positive results during the last two weeks?	
**Prescribed drugs**	
5. How many patients have been prescribed Artemether-Lumefantrine for 5-14 kg in the last two weeks? *(999=don't know)*	
6. How many patients have been prescribed Artemether-Lumefantrine for 15-24 kg in the last two weeks?	
7. How many patients have been prescribed Artemether-Lumefantrine for 25-34 kg in the last two weeks?	
8. How many patients have been prescribed Artemether-Lumefantrine for >35 kg in the last two weeks?	
9. How many patients have been prescribed injections of Artemether IM in the last two weeks?	
10. How many patients have been prescribed injections of Artesunate IV in the last two weeks?	
11. How many patients have been prescribed Artesunate suppositories in the last two weeks?	
12. How many patients have been prescribed Artesunate combination in the last two weeks?	
13. How many patients have been prescribed Dihydroartemisinin in the last two weeks?	
14. How many patients have been prescribed injections of Artemisinin IV in the last two weeks?	
15. How many patients have been prescribed Artemisinin combinations in the last two weeks?	
16. How many patients have been prescribed other ACT in the last two weeks?	
17. How many patients have been prescribed Amodiaquine in tablets in the last two weeks?	

HEALTH FACILITY SURVEY – HF STOCKS

REGISTER BOOK FORM

18. How many patients have been prescribed Sulfadoxine-Pyrimethamine in the last two weeks?	
19. How many patients have been prescribed Mefloquine in the last two weeks?	
20. How many patients have been prescribed Quinine in tablets in the last two weeks?	
21. How many patients have been prescribed injections of Quinine IV in the last two weeks?	
22. How many patients have been prescribed Atovaquone-Proguanil in the last two weeks?	
23 (a). Is there any other antimalarial that has not been listed?	❑ Yes ❑ No ❑ Don't know
23 (b). If yes, what is the name of the antimalarial? *(Write the brand name)*	....
23 (c). How many patients have been prescribed this antimalarial in the last two weeks?	Number:
24 (a). Is there any other antimalarial that has not been listed?	❑ Yes ❑ No ❑ Don't know
24 (b). If yes, what is the name of the antimalarial? *(Write the brand name)*	....
24 (c). How many patients have been prescribed this antimalarial in the last two weeks?	Number:
25. How many patients have been prescribed Amoxicillin in the last two weeks?	
26. How many patients have been prescribed Cotrimoxazole in the last two weeks?	
27. How many patients have been prescribed Doxycycline in the last two weeks?	
28. How many patients have been prescribed Cloxacilline in the last two weeks?	
**Distributed drugs**	
29. How many patients have been distributed Artemether-Lumefantrine for 5-14 kg in the last two weeks?	
30. How many patients have been distributed Artemether-Lumefantrine for 15-24 kg in the last two weeks?	
31. How many patients have been distributed Artemether-Lumefantrine for 25-34 kg in the last two weeks?	
32. How many patients have been distributed Artemether-Lumefantrine for >35 kg in the last two weeks?	
33. How many patients have been distributed injections of Artemether IM in the last two weeks?	
34. How many patients have been distributed injections of Artesunate IV in the last two weeks?	
35. How many patients have been distributed Artesunate suppositories in the last two weeks?	
36. How many patients have been distributed Artesunate combination in the last two weeks?	
37. How many patients have been distributed Dihydroartemisinin in the last two weeks?	
38. How many patients have been distributed injections of Artemisinin IV in the last two weeks?	
39. How many patients have been distributed Artemisinin combinations in the last two weeks?	
40. How many patients have been distributed other ACT in the last two weeks?	
41. How many patients have been distributed Amodiaquine in tablets in the last two weeks?	
42. How many patients have been distributed Sulfadoxine-Pyrimethamine in the last two weeks?	
43. How many patients have been distributed Mefloquine in the last two weeks?	
44. How many patients have been distributed Quinine in tablets in the last two weeks?	
45. How many patients have been distributed injections of Quinine IV in the last two weeks?	
46. How many patients have been distributed Atovaquone-Proguanil in the last two weeks?	
47 (a). Is there any other antimalarial that has not been listed?	❑ Yes ❑ No ❑ Don't know
47 (b). If yes, what is the name of the antimalarial? *(Write the brand name)*	....
47 (c). How many patients have been distributed this antimalarial in the last two weeks?	Number:

HEALTH FACILITY SURVEY – HF STOCKS

REGISTER BOOK FORM

48 (a). Is there any other antimalarial that has not been listed?	❑ Yes ❑ No ❑ Don't know
48 (b). If yes, what is the name of the antimalarial? *(Write the brand name)*	....
48 (c). How many patients have been distributed this antimalarial in the last two weeks?	Number:
49. How many patients have been distributed Amoxicillin in the last two weeks?	
50. How many patients have been distributed Cotrimoxazole in the last two weeks?	
51. How many patients have been distributed Doxycycline in the last two weeks?	
52. How many patients have been distributed Cloxacilline in the last two weeks?	

OUTLET SURVEY

STOCKS FORM

General information:

Staff initials: |__|__|__|

Date of survey (Day/Month/Year): |__|__ / |__|__ / |__|__||__|__|

Region: .... District: ....

Ward.. Outlet Name ....

Altitude: Latitude: Longitude: ....

Type of outlet: ❑ ADDO ❑ Duka la dawa baridi ❑ Pharmacy ❑ Kiosk, general store

EQUIPMENT AND SUPPLIES FOR DIAGNOSTIC TESTING

Which of the following were observed at the health facility in or near at least one consulation room? (make sure you have seen!!)

1. At least 25 RDTs (not expired) ❑ Yes ❑ No ❑ Don't know
2. Are there any mRDTs in stock today (not expired)? ❑ Yes ❑ No ❑ Don't know
3. Have mRDTs been in stock every day for the last three months, according to log books or stock cards? ❑ Yes ❑ No ❑ Don't know

STOCKS OF DRUGS

4. Is **CoartemD** for **5-14 kg** in stock today?	❑ Yes ❑ No ❑ Don't know
5. Are there at least 20 treatments (not expired) in stock today?	❑ Yes ❑ No ❑ Don't know
6. Was this drug in stock every day for the last three months, according to log books or stock cards?	❑ Yes ❑ No ❑ Don't know
7. Is **CoartemD** for **15-24 kg** in stock today?	❑ Yes ❑ No ❑ Don't know
8. Are there at least 20 treatments (not expired) in stock today?	❑ Yes ❑ No ❑ Don't know
9. Was this drug in stock every day for the last three months, according to log books or stock cards?	❑ Yes ❑ No ❑ Don't know
10. Is **Alu blister** for **5-14 kg** in stock today?	❑ Yes ❑ No ❑ Don't know
11. Are there at least 20 blister packs (not expired) in stock today?	❑ Yes ❑ No ❑ Don't know
12. Was this drug in stock every day for the last three months, according to log books or stock cards?	❑ Yes ❑ No ❑ Don't know
13. Is **Alu blister** pack for **15-24 kg** in stock today?	❑ Yes ❑ No ❑ Don't know
14. Are there at least 20 blister packs (not expired) in stock today?	❑ Yes ❑ No ❑ Don't know

OUTLET SURVEY

STOCKS FORM

15. Was this drug in stock every day for the last three months, according to log books or stock cards?	❑ Yes ❑ No ❑ Don't know
16. Is **Alu blister** pack for **25-34 kg** in stock today?<br>17. Are there at least 20 blister packs (not expired) in stock today?<br>18. Was this drug in stock every day for the last three months, according to log books or stock cards?	❑ Yes ❑ No ❑ Don't know<br>❑ Yes ❑ No ❑ Don't know<br>❑ Yes ❑ No ❑ Don't know
19. Is **Alu blister** pack for **> 35 kg** in stock today?<br>20. Are there at least 20 blister packs (not expired) in stock today?<br>21. Was this drug in stock every day for the last three months, according to log books or stock cards?	❑ Yes ❑ No ❑ Don't know<br>❑ Yes ❑ No ❑ Don't know<br>❑ Yes ❑ No ❑ Don't know
22. Is **Artesunate** tablets in stock today?<br>23. Are there at least 20 blister packs (not expired) in stock today?<br>24. Was this drug in stock every day for the last three months, according to log books or stock cards?	❑ Yes ❑ No ❑ Don't know<br>❑ Yes ❑ No ❑ Don't know<br>❑ Yes ❑ No ❑ Don't know
25. Is **Artesunate-amodiaquine** tablets in stock today?<br>26. Are there at least 20 blister packs (not expired) in stock today?<br>27. Was this drug in stock every day for the last three months, according to log books or stock cards?	❑ Yes ❑ No ❑ Don't know<br>❑ Yes ❑ No ❑ Don't know<br>❑ Yes ❑ No ❑ Don't know
28. Is **Artemether (IM injectable)** in stock today?<br>29. Are there at least 20 blister packs (not expired) in stock today?<br>30. Was this drug in stock every day for the last three months, according to log books or stock cards?	❑ Yes ❑ No ❑ Don't know<br>❑ Yes ❑ No ❑ Don't know<br>❑ Yes ❑ No ❑ Don't know
31. Is **Artesunate (IV injectable)** in stock today?<br>32. Are there at least 20 blister packs (not expired) in stock today?<br>33. Was this drug in stock every day for the last three months, according to log books or stock cards?	❑ Yes ❑ No ❑ Don't know<br>❑ Yes ❑ No ❑ Don't know<br>❑ Yes ❑ No ❑ Don't know
34. Is **Artemisinin suppositories** in stock today?<br>35. Are there at least 20 blister packs (not expired) in stock today?<br>36. Was this drug in stock every day for the last three months, according to log books or stock cards?	❑ Yes ❑ No ❑ Don't know<br>❑ Yes ❑ No ❑ Don't know<br>❑ Yes ❑ No ❑ Don't know
37. Are there other **Artemisinin monotherapy** in stock today?<br>38. Are there at least 20 blister packs (not expired) in stock today?<br>39. Was this drug in stock every day for the last three months, according to log books or stock cards?	❑ Yes ❑ No ❑ Don't know<br>❑ Yes ❑ No ❑ Don't know<br>❑ Yes ❑ No ❑ Don't know

OUTLET SURVEY

STOCKS FORM

40. Is **Artesunate suppositories** in stock today?	❑ Yes ❑ No ❑ Don't know
41. Are there at least 20 blister packs (not expired) in stock today?	❑ Yes ❑ No ❑ Don't know
42. Was this drug in stock every day for the last three months, according to log books or stock cards?	❑ Yes ❑ No ❑ Don't know
43. Are there **other ACTs** in stock today?	❑ Yes ❑ No ❑ Don't know
44. Are there at least 20 blister packs (not expired) in stock today?	❑ Yes ❑ No ❑ Don't know
45. Was this drug in stock every day for the last three months, according to log books or stock cards?	❑ Yes ❑ No ❑ Don't know
46. Are **Amodiaquine** tablets in stock today?	❑ Yes ❑ No ❑ Don't know
47. Are there at least 20 blister packs (not expired) in stock today?	❑ Yes ❑ No ❑ Don't know
48. Was this drug in stock every day for the last three months, according to log books or stock cards?	❑ Yes ❑ No ❑ Don't know
49. Are **Mefloquine** tablets in stock today?	❑ Yes ❑ No ❑ Don't know
50. Are there at least 20 blister packs (not expired) in stock today?	❑ Yes ❑ No ❑ Don't know
51. Was this drug in stock every day for the last three months, according to log books or stock cards?	❑ Yes ❑ No ❑ Don't know
52. Are **SP/Fansidar** tablets in stock today?	❑ Yes ❑ No ❑ Don't know
53. Are there at least 20 blister packs (not expired) in stock today?	❑ Yes ❑ No ❑ Don't know
54. Was this drug in stock every day for the last three months, according to log books or stock cards?	❑ Yes ❑ No ❑ Don't know
55. Is **Quinine** tablets in stock today?	❑ Yes ❑ No ❑ Don't know
56. Are there at least 20 blister packs (not expired) in stock today?	❑ Yes ❑ No ❑ Don't know
57. Was this drug in stock every day for the last three months, according to log books or stock cards?	❑ Yes ❑ No ❑ Don't know
58. Is **Quinine** or **Quinidine (injectable)** in stock today?	❑ Yes ❑ No ❑ Don't know
59. Are there at least 20 blister packs (not expired) in stock today?	❑ Yes ❑ No ❑ Don't know
60. Was this drug in stock every day for the last three months, according to log books or stock cards?	❑ Yes ❑ No ❑ Don't know
61. Is **Amoxicillin** in stock today?	❑ Yes ❑ No ❑ Don't know
62. Are there at least 20 blister packs (not expired) in stock today?	❑ Yes ❑ No ❑ Don't know
63. Was this drug in stock every day for the last three months, according to log books or stock cards?	❑ Yes ❑ No ❑ Don't know
64. Is **Cotrimoxazole** in stock today?	❑ Yes ❑ No ❑ Don't know
65. Are there at least 20 blister packs (not expired) in stock today?	❑ Yes ❑ No ❑ Don't know

OUTLET SURVEY

STOCKS FORM

66. Was this drug in stock every day for the last three months, according to log books or stock cards?	❑ Yes ❑ No ❑ Don't know
67. Are there **other antibiotics** in stock today?<br>68. Are there at least 20 blister packs (not expired) in stock today?<br>69. Was this drug in stock every day for the last three months, according to log books or stock cards?	❑ Yes ❑ No ❑ Don't know<br>❑ Yes ❑ No ❑ Don't know<br>❑ Yes ❑ No ❑ Don't know
70. Are there **antipyretics** in stock today?<br>71. Are there at least 20 blister packs (not expired) in stock today?<br>72. Was this drug in stock every day for the last three months, according to log books or stock cards?	❑ Yes ❑ No ❑ Don't know<br>❑ Yes ❑ No ❑ Don't know<br>❑ Yes ❑ No ❑ Don't know
73. Are there **traditional herbs** in stock today?<br>74. Are there at least 20 packs (not expired) in stock today?<br>75. Was this drug in stock every day for the last three months, according to log books or stock cards?	❑ Yes ❑ No ❑ Don't know<br>❑ Yes ❑ No ❑ Don't know<br>❑ Yes ❑ No ❑ Don't know

Swiss TPH
Swiss Tropical and Public Health Institute
Schweizerisches Tropen- und Public Health-Institut
Institut Tropical et de Santé Publique Suisse

AMACO Project

IFAKARA HEALTH INSTITUTE
research | training | services

OUTLET SURVEY

REGISTER BOOK FORM

General information:

Staff initials: |__|__|__|

Date of survey (Day/Month/Year): |__|__ / |__|__ / |__|__||__|__|

Region: .... District: ....

Ward .... Outlet Name...

Altitude: Latitude: Longitude: ....

Type of outlet: ❑ ADDO ❑ Duka la dawa baridi ❑ Pharmacy ❑ Kiosk, general store

PHARMACY REGISTER BOOK

**Visits**	
1. How many visits have occured during the last two weeks?	
**Malaria tests**	
2. How many mRDTs were done during the last two weeks?	
**Positive tests**	
3. How many mRDTs were positive during the last two weeks?	
**Distributed drugs**	
4. How many patients have been distributed Artemether-Lumefantrine for 5-14 kg in the last two weeks?	
5. How many patients have been distributed Artemether-Lumefantrine for 15-24 kg in the last two weeks?	
6. How many patients have been distributed Artemether-Lumefantrine for 25-34 kg in the last two weeks?	
7. How many patients have been distributed Artemether-Lumefantrine for >35 kg in the last two weeks?	
8. How many patients have been distributed injections of Artemether IM in the last two weeks?	
9. How many patients have been distributed injections of Artesunate IV in the last two weeks?	
10. How many patients have been distributed Artesunate suppositories in the last two weeks?	
11. How many patients have been distributed Artesunate combination in the last two weeks?	
12. How many patients have been distributed Dihydroartemisinin in the last two weeks?	
13. How many patients have been distributed injections of Artemisinin IV in the last two weeks?	
14. How many patients have been distributed Artemisinin combinations in the last two weeks?	
15. How many patients have been distributed other ACT in the last two weeks?	
16. How many patients have been distributed Amodiaquine in tablets in the last two weeks?	
17. How many patients have been distributed Sulfadoxine-Pyrimethamine in the last two weeks?	
18. How many patients have been distributed Mefloquine in the last two weeks?	
19. How many patients have been distributed Quinine in tablets in the last two weeks?	
20. How many patients have been distributed injections of Quinine IV in the last two weeks?	
21. How many patients have been distributed Atovaquone-Proguanil in the last two weeks?	

OUTLET SURVEY

REGISTER BOOK FORM

22 (a). Is there any other antimalarial that has not been listed?	❑ Yes ❑ No ❑ Don't know
22 (b). If yes, what is the name of the antimalarial? *(Write the brand name)*	....
22 (c). How many patients have been distributed this antimalarial in the last two weeks?	Number:
23 (a). Is there any other antimalarial that has not been listed?	❑ Yes ❑ No ❑ Don't know
23 (b). If yes, what is the name of the antimalarial? *(Write the brand name)*	....
23 (c). How many patients have been distributed this antimalarial in the last two weeks?	Number:
24. How many patients have been distributed Amoxicillin in the last two weeks?	
25. How many patients have been distributed Cotrimoxazole in the last two weeks?	
26. How many patients have been distributed Doxycycline in the last two weeks?	
27. How many patients have been distributed Cloxacilline in the last two weeks?	
